# Mitigation Measures for Pandemic Influenza in Italy: An Individual Based Model Considering Different Scenarios

**DOI:** 10.1371/journal.pone.0001790

**Published:** 2008-03-12

**Authors:** Marta Luisa Ciofi degli Atti, Stefano Merler, Caterina Rizzo, Marco Ajelli, Marco Massari, Piero Manfredi, Cesare Furlanello, Gianpaolo Scalia Tomba, Mimmo Iannelli

**Affiliations:** 1 National Center for Epidemiology Surveillance and Health Promotion, Istituto Superiore di Sanità, Rome, Italy; 2 Fondazione Bruno Kessler, Trento, Italy; 3 Department of Pharmaco-Biology, University of Bari, Bari, Italy; 4 Department of Information Engineering and Computer Science, University of Trento, Trento Italy; 5 Department of Statistics and Mathematics Applied to Economics, University of Pisa, Pisa, Italy; 6 Department of Mathematics, University of ‘Tor Vergata’, Roma, Italy; 7 Department of Mathematics, University of Trento, Trento, Italy; Centre for DNA Fingerprinting and Diagnostics, India

## Abstract

**Background:**

Individual-based models can provide the most reliable estimates of the spread of infectious diseases. In the present study, we evaluated the diffusion of pandemic influenza in Italy and the impact of various control measures, coupling a global SEIR model for importation of cases with an individual based model (IBM) describing the Italian epidemic.

**Methodology/Principal Findings:**

We co-located the Italian population (57 million inhabitants) to households, schools and workplaces and we assigned travel destinations to match the 2001 census data. We considered different R_0 _values (1.4; 1.7; 2), evaluating the impact of control measures (vaccination, antiviral prophylaxis -AVP-, international air travel restrictions and increased social distancing). The administration of two vaccine doses was considered, assuming that first dose would be administered 1-6 months after the first world case, and different values for vaccine effectiveness (VE). With no interventions, importation would occur 37–77 days after the first world case. Air travel restrictions would delay the importation of the pandemic by 7–37 days. With an R_0 _of 1.4 or 1.7, the use of combined measures would reduce clinical attack rates (AR) from 21–31% to 0.3–4%. Assuming an R_0_ of 2, the AR would decrease from 38% to 8%, yet only if vaccination were started within 2 months of the first world case, in combination with a 90% reduction in international air traffic, closure of schools/workplaces for 4 weeks and AVP of household and school/work close contacts of clinical cases. Varying VE would not substantially affect the results.

**Conclusions:**

This IBM, which is based on country-specific demographic data, could be suitable for the real-time evaluation of measures to be undertaken in the event of the emergence of a new pandemic influenza virus. All preventive measures considered should be implemented to mitigate the pandemic.

## Introduction

The emergence of the highly virulent A/H5N1 avian influenza strain [1], which is capable of infecting humans [2] and could acquire the capacity for efficient person-to-person transmission, has given rise to concerns over the risk of a future influenza pandemic [3]. In fact, this virus, or a closely related one, is considered to be the leading contender as the source of the next human influenza pandemic [4]–[6]. For these reasons, countries have been urged to strengthen their preparedness plans [6], and several countries have considered stockpiling both antiviral drugs and monovalent influenza vaccines containing potentially pandemic strains, such as A/H5N1 (i.e., a pre-pandemic vaccine), for population priming [7].

However, some control measures can be costly (e.g., stockpiling antiviral drugs, vaccines, and a pre-pandemic vaccine), and others could have limited social acceptance (e.g., closure of schools/workplaces and travel restrictions). For these reasons, several countries have used mathematical models to predict the spread of infection at the national level, which is an important aspect of preparedness, and to evaluate the feasibility of containing the pandemic using different strategies [8]–[14].

Individual-based models can provide the most reliable estimates of the spread of influenza [8]–[11]. In the present study, we evaluated the diffusion of pandemic influenza in Italy and the impact of various control measures, coupling a global SEIR model with an individual based model. We used actual demographic data, obtained from the 2001 census, which allowed us to simulate the spread of an influenza pandemic and the impact of control measures. In particular, we examined the impact of antiviral prophylaxis of close contacts, social distancing measures, international air travel restrictions, and vaccination (both pandemic and pre-pandemic vaccine), under different R_0_ values. Since it has been shown that seasonal influenza vaccine effectiveness is higher in adults than in elderly persons and children [15]–[17], we also assumed that both pandemic and pre-pandemic vaccine effectiveness would vary by age.

## Methods

The worldwide spread of pandemic influenza and the consequent importation of cases in Italy were modelled using a global deterministic SEIR (susceptible–exposed, but not yet infectious-infectious–recovered, and no longer susceptible) model (herein referred to as the “global SEIR model”) [14]. The national impact of an influenza pandemic in Italy and of various control measures was predicted using a stochastic individual-based SEIR model (IBM) [8]–[11].

In both models, we assumed that the latency period for influenza was the same as the incubation period: duration of 1.5 (±0.5 SD) days. In the IBM, we assumed that the duration of infectiousness varied over time, as a lognormal function [8], [9]. Infectiousness peaked at 1.75 days, and its duration was truncated at 10 days [8], [9]. This corresponded to an average generation time of 2.6 days. In the global SEIR model, the infectious period was assumed to be constant over time and was set at 1.5 days [13], [18], to give the same growth rate as the IBM [9] (See Text S1).

In both the SEIR and IBM models, we considered different transmission rates to obtain R_0_ values of 1.4, 1.7, and 2, which in the IBM corresponded to cumulative clinical attack rates (AR) of 21.2%, 30.8%, and 38.7%, respectively, indicating a mild, moderate and severe scenario [19]. The results were obtained by averaging 50 simulations for each scenario.

### Global SEIR model

In this model, we assumed that infectious individuals were all symptomatic and no longer travelling and that exposed individuals were asymptomatic and possibly travelling before the infectious phase. We coupled the results of the global SEIR model with 2003 data on arrivals and departures in Italy's 38 international airports [20]. Specifically, the number of imported exposed individuals was expressed as 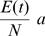, where *E(t)* is the number of exposed individuals obtained with the global SEIR model at time *t*, *N* is the world population, and *a* is the total number of persons arriving daily in Italy (an average of 70,000). The probability of importing infections to municipalities with international airports is given by 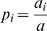, where *a_i_* is the number of persons arriving daily in the *i-*th airport (ranging from 0.1 to 22,000 on average) (see Text S1).

National data on in-coming flow by land and sea were not easily available, and were therefore not included in the model.

### IBM

#### Socio-demographic structure

Data on Italy's population were obtained from the 2001 census, which includes information on age structure, household size, household composition (e.g., single individuals or couples with or without children), school attendance, employment categories, municipality of residence, and data on the population that commutes daily within national borders (see Text S1) [21]. The 2001 census was performed by collecting data through direct interviews with individuals with official declared residence in a given municipality and those actually living in that municipality.

In the model, which can be viewed as a patch model with multiple levels of mixing, Italy's 56,995,744 inhabitants were hierarchically grouped by municipality (n = 8,101), province (n = 103) and region (n = 20) (Figure S1). The mean radius of Italian municipalities, provinces and regions is, respectively, 3.02 km (SD = 1.64 km), 13.01 km (SD = 3.56 km), and 67.01 km (SD = 18.32 km).

Individuals were randomly placed in households to match the 2001 census data on age structure and on household size and composition (Figure S2). Nine different types of households were considered (e.g., singles or couples, with or without children, with or without additional additional members, adults living together) and individuals were co-located in households according to specific data on the percentage of the different household types, their size, the age of the household head (see Text S1, Tables S1, S2, S3, S4, S5, Figure S3).

Children and adolescents aged 0–18 years were assigned to one of six levels of school (i.e., from day care to university) (Table 1) (see Text S1).

**Table 1:** School/workplace attendance and contact patterns

School category	Age group in years	% attending population	Average number of close contacts	% of clinical cases staying at home
Day care	0–2	14	20	90
Nursery school/Kindergarten	3–5	90	40	90
Primary school	6–10	97	19	80
Middle school	11–13	96	21	80
High school	14–18	82	21	75
University	19–24	31	34	50
Workplace	≥15	43	5	50

In individuals aged ≥15 years, the average employment rate was 43%, ranging from 1.5% for 15 year-olds to 73% for individuals aged 36 years [22]. Each working individual was randomly assigned to one of seven employment categories, defined by the number of employees in the workplace (1–5, 6–9, 10–19, 20–49, 50–99, 100–249, and ≥250) [21] (see Text S1, Figure S2). In the model, schools and workplaces were located at the centroid of each municipality.

We modelled travel destinations using data on commuting, which were available from the 2001 census for persons ≥15 years of age [21]. The census includes individual data on daily commutes to school or work, specifiying whether the commute is within the same municipality of residence, outside the municipality but within the same province, outside the province but within the same region, or outside of the region. We used these data to develop a gravity model [22], in which the probability of commuting from one municipality to another increases with the population sizes and decreases with the distance. Moreover, the employed gravity model accounts for the spatial variability in the proportion of commuters (see Text S1, Figures S4 and S5).

Schools and workplaces were generated using previously reported methods [8]. Briefly, we used national statistics on the average size of schools and workplaces [23] to generate a synthetic population of schools and workplaces distributed in space with a density proportional to the local population density. These methods allocate students (including those <15 years of age) to schools and workers to workplaces using census data (see Text S1). Moreover, students and workers were clustered to form groups of persons in close contact (i.e., classes for schools and groups of colleagues for workplaces). For schools, the average number of persons in a class, by school category (e.g., daycare, nursery school, elementary school) were determined based on available data (Table 1) [23].

#### Transmission model

Infection can be transmitted within households and schools/workplaces and among random contacts. For a given individual, the probability of being infected is 1−*e* ^−*λT*^, where *λ* is the sum of the force of infections coming from the three above-mentioned sources and *T* is the time-step of the simulation, which is fixed at 0.25 days.

Whereas we assumed homogeneous mixing in households, schools and workplaces, all susceptible individuals are considered as random contacts of an infectious individual, and the probability of being infected is weighted by a kernel function which is a decreasing function of the distance [8], [9]. The parameters of the kernel were optimized on the basis of the distance to work/school distribution as resulting by the application of the gravity model employed for assigning travel destinations (see Text S1).

In the three scenarios considered (R_0_ values of 1.4, 1.7, and 2) [19], different values of the transmission rates were determined for the different transmission sources (households, schools/workplaces and random contacts) (see Text S1).

The basic reproductive number (R_0_) of the simulated epidemics was estimated according to a previously published model [8] (See Text S1, Figure S6). We also assumed that 50% of infections result in clinical illness and that the transmission rate does not differ between symptomatic and asymptomatic individuals [9].

All the simulations were run until the incidence was zero.

#### Control measures

We considered the following control measures: a) vaccination; b) antiviral prophylaxis (AVP), c) social distancing, and d) air travel restrictions.

*Vaccination.* The target population was divided into 4 categories: i) personnel providing essential services (15% of the 25–60-year-old working population) [21]; ii) elderly persons (≥65 years of age); iii) children and adolescents from 2 to 18 years of age; and iv) adults from 40 to 64 years of age. Vaccination was modelled by reducing the proportion of susceptible individuals in the target population. This proportion depends on vaccination coverage (VC) and vaccine effectiveness (VE). We assumed that vaccination consists of two vaccine doses administered one month apart and that VC was 60% of the target population. This VC was chosen on the basis of the 2005–2006 seasonal influenza coverage, which was 68% in elderly persons (>64 years) [24]. We assumed that one week is necessary for administering each vaccine dose to all target categories. Vaccination was considered to be effective beginning 15 days after the administration of the second dose. Three different assumptions on VE were considered: i) VE of 70%, for all age-groups; ii) VE of 50%, for all age-groups; and iii) VE of 59% for individuals aged 2–18 years [16], 70% for individuals aged 40–64 years [15], and 40% for individuals aged ≥65 years [17]. We assumed that individuals are vaccinated irrespective of whether or not they were infectious or ill.

When considering the impact of single interventions, we assumed that vaccination begins 1, 2, 3, 4, 5 or 6 months after the first world case, targeting three of the target categories (i.e., personnel providing essential services, elderly persons, and 2–18 year-olds), and assuming a VE of 70% for all three categories. When considering multiple interventions, we assumed that vaccination begins at 2, 3 or 4 months after the first world case, and we considered the different assumptions for VE reported above (70% for all, 50% for all, or varying by age).

Given that an estimated 3–6 months would be required to produce pandemic influenza vaccines, the administration of a first dose within 3 months of the first world case would be possible only if this dose contained a precursor of the pandemic strain [4], followed by a dose of pandemic vaccine. The actual VE of this regimen was assumed to be equal to that of two doses of the pandemic vaccine.

*Antiviral*. We took into consideration the administration of one course of antiviral drugs, providing therapy for the index case and prophylaxis for close contacts. Both therapy and prophylaxis were assumed to start one day after clinical onset in the index case. The treatment of the index case was assumed to reduce infectiousness by 70% [8]–[11], whereas AVP was assumed to reduce susceptibility to infection by 30%, infectiousness by 70%, and the occurrence of symptomatic disease by 60% [10].

We assumed that AVP be provided to 90% of the close contacts of clinical cases (50% of all infected individuals), with a treatment course of 10 days [25]. Two different definitions of close contacts were used: i) household contacts only; and ii) household contacts plus close contacts in the school or workplace (Table 1). We considered administering AVP for the entire epidemic period; however, since the feasibility of actually doing this would be limited, we also considered adminstering AVP as a policy to be used only for the first 8 weeks after the occurrence of the first Italian case.

*Social distancing*. We considered the nationwide closing of all schools and some public offices not providing essential services, corresponding to 20% of all employees in these types of offices [26]. We assumed that school and office closings begin 4 weeks after the onset of the first 20 symptomatic cases in Italy and that this measure be maintained for 4 weeks.

We also assumed that symptomatic individuals spontaneously limit their school/work attendance. The proportion of symptomatic individuals staying at home from school/workplace would vary by age, from 90% among children <6 years of age to 50% among the working population (Table 1).

d) *Air travel restrictions*. We considered travel restrictions that would reduce incoming international flights by 90% or 99%, starting from day 30 of the first world case [9] and lasting for the entire duration of the epidemic, or until two months after the introduction of the first case in Italy. The reduction of domestic air travel and the control of land and sea borders were not considered in the model.

## Results

### Baseline dynamics

**Figure 1:**
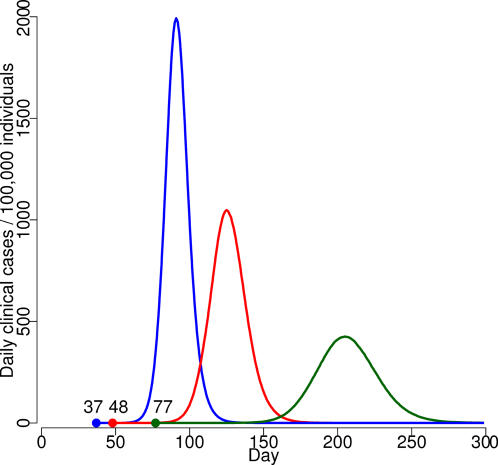
Baseline simulations under different R_0_ scenarios (blue line: R_0_ = 2; red line: R_0_ = 1.7; green line: R_0_ = 1.4). Bullet points represent the first Italian case and the time elapsed from the first world case.

For different R_0_ scenarios, the results of the global SEIR model showed that the number of imported symptomatic cases would be 53,000, 72,000, and 83,000 (See Text S1, Figure S7), with the first Italian case appearing, respectively, after 77, 48 and 37 days (Figure S8); the epidemic curves for these scenarios are shown in Figure 1.

**Figure 2:**
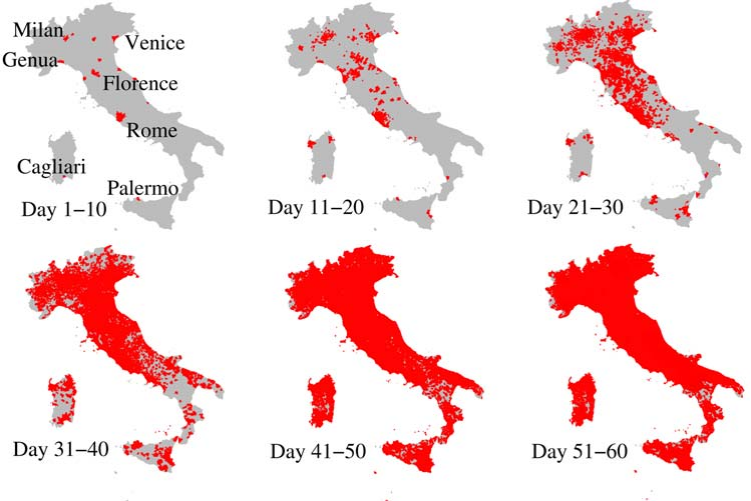
Spatial spread of pandemic influenza in Italy, R_0_ = 1.7. Red areas represent municipalities where at least one case is present.

For R_0_ = 1.7, the spatial spread of the epidemic showed that for the first 10 days the new cases would be confined to the municipalities where cases were first imported (Figure 2). At 11–20 days, new cases would begin to occur far from these municipalities, mainly in municipalities with a large population. At 21–40 days (the exponential growth phase), infection would spread simultaneously to nearly the entire country, with no clear spatial pattern.

The epidemic peak is reached after 202, 125 and 91 days, respectively, for the three different scenarios (Figure 1). The pandemic season at the national level would last for a period of 3 to 6 months, with an average of 67,000–243,000 clinical cases per day. The cumulative infected AR would be 42.4%, 61.6% and 77.4%, for the three scenarios, corresponding to a clinical AR of 21.2%, 30.8%, and 38.7%. The clinical daily-peak AR would be 0.4%, 1.0% and 1.9%, respectively. Only the clinical AR is considered below.

The comparisons of baseline scenarios with historical data are described in the Text S1 (Figures S9 and S10).

### Impact of control measures

#### Single measures

**Figure 3:**
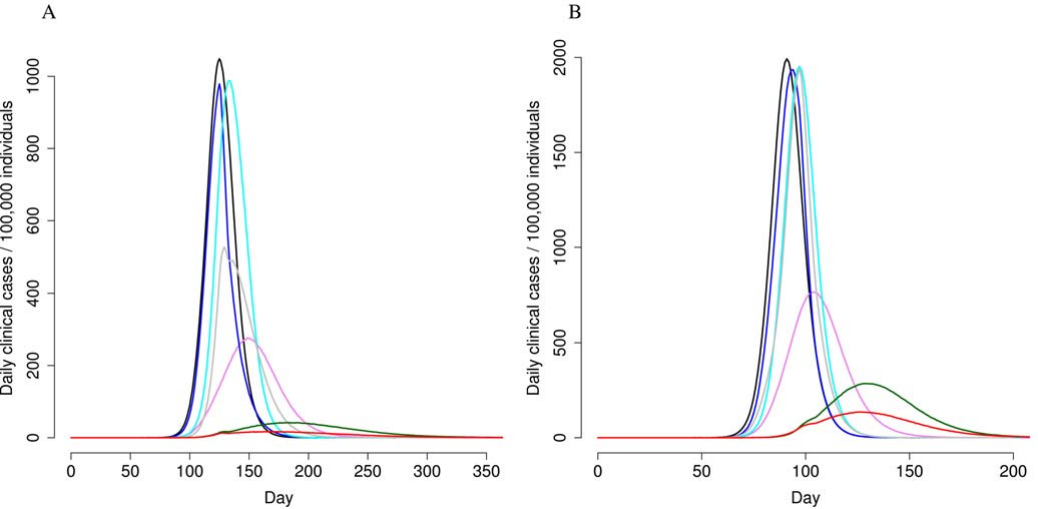
Clinical AR, by control measure and scenario (Panel A: R_0_ = 1.7; Panel B: R_0_ = 2). black = baseline results; light blue =  90% air travel restriction; violet = AVP to household contacts; blue = vaccination, administering first dose within 3 months of the first world case for R_0_ = 1.7, or within 2 months for R_0_ = 2; grey = 90% air travel restriction+vaccination, as reported for the blue line; green = all control measures combined; red = all control measures combined, extending AVP to school/work close contacts.

The results of the single control measures for different scenarios are reported in Tables 2–3. International air travel restriction would not affect the AR (Table 2) but could delay the importation of cases, increasing the time elapsed from the first world case to importation from a minimum of 7 days to a maximum of 37 days, depending on the R_0_ and the level of restriction (Table 3, Figure S8). The pandemic peak would also be delayed by 6–39 days (Table 2; Figure 3). Nationwide closure of schools and workplaces not providing essential services would delay the time of occurrence of the peak by 5–8 days, depending from the scenario considered.

**Table 2:** Clinical attack rates and peak day, by scenario and control measure.

Control measure	Scenario											
	Mild				Moderate				Severe			
	Cumulative AR [%] (95% CI)	Peak day (95% CI)	Peak daily AR [%] (95% CI)	Million courses	Cumulative AR [%] (95% CI)	Peak day (95% CI)	Peak daily AR [%] (95% CI)	Million courses	Cumulative AR [%] (95% CI)	Peak day (95% CI)	Peak daily AR [%] (95% CI)	Million courses
**None**	21.2 (21.13–21.27)	202 (200–204)	0.42 (0.41–0.43)	-	30.81 (30.73–30.90)	125 (123–126)	1.03 (1.02–1.05)	-	38.69 (38.60–38.77)	91 (89–92)	1.93 (1.87–1.97)	-
**Air travel restriction**												
* 90%*	21.16 (21.08–21.24)	225 (223–228)	0.42 (0.42–0.43)	-	30.81 (30.74–30.88)	135 (133–138)	1.01 (0.99–1.03)	-	38.72 (38.62–38.80)	97 (95–100)	1.90 (1.86–1.93)	-
* 99%*	21.14 (21.05–21.23)	241 (233–245)	0.40 (0.38–0.41)	-	30.82 (30.75–30.88)	150 (146–152)	1.03 (0.97–1.10)	-	38.72 (38.62–38.81)	108 (106–109)	1.91 (1.84–1.94)	-
**Social distancing**												
* closure of schools and workplaces for 4 weeks*	21.18 (21.06–21.26)	207 (205–211)	0.43 (0.43–0.43)	-	30.81 (30.70–30.88)	132 (130–135)	1.03 (1.02–1.04)	-	38.68 (38.60–38.75)	99 (97–102)	1.91 (1.89–1.94)	-
**Antiviral prophylaxis**												
* Household close contacts*	4.58 (4.46–4.69)	255 (251–263)	0.03 (0.03–0.03)	7,3	15.47 (15.39–15.55)	150 (149–151)	0.27 (0.27–0.28)	23,5	24.91 (24.83–24.98)	104 (100–106)	0.76 (0.75–0.77)	35,4
**Vaccination, by interval from the first world case**												
* 1 month*	7.44 (7.36–7.54)	238 (235–239)	0.07 (0.07–0.07)	14,4	17.81 (17.72–17.92)	144 (141–146)	0.36 (0.36–0.37)	14,4	27.12 (27.06–27.20)	99 (97–100)	0.94 (0.94–0.96)	14,4
* 2 months*	7.47 (7.40–7.57)	237 (234–241)	0.07 (0.07–0.07)	14,4	18.07 (17.97–18.14)	133 (132–134)	0.36 (0.36–0.37)	14,4	36.29 (36.10–36.52)	92 (89–93)	1.91 (1.90–1.94)	14,4
* 3 months*	7.48 (7.39–7.61)	235 (233–238)	0.07 (0.07–0.07)	14,4	25.48 (24.84–25.94)	125 (122–126)	1.0 (0.98–1.01)	14,4	38.67 (38.58–38.75)	92 (89–93)	1.91 (1.90–1.94)	14,4
* 4 months*	7.66 (7.60–7.74)	218 (211–222)	0.08 (0.08–0.08)	14,4	30.58 (30.53–30.69)	125 (124–127)	1.0 (0.98–1.01)	14,4	38.68 (38.58–38.76)	92 (89–93)	1.91 (1.90–1.94)	14,4
* 5 months*	10.46 (9.9–11.19)	182 (178–186)	0.24 (0.20–0.28)	14,4	30.80 (30.70–30.89)	125 (124–127)	1.0 (0.98–1.01)	14,4	38.68 (38.60–38.77)	92 (89–93)	1.91 (1.90–1.94)	14,4
* 6 months*	17.51 (16.87–18.29)	202 (200–203)	0.43 (0.43–0.44)	14,4	30.81 (30.72–30.89)	125 (124–127)	1.0 (0.98–1.01)	14,4	38.68 (38.59–38.78)	92 (89–93)	1.91 (1.90–1.94)	14,4

VE = 70%; vaccination target categories: personnel providing essential services, elderly persons, individuals 2–18 years of age

**Table 3:** Interval (in days) from the first world case to the importation of the first case in Italy, by scenario and level of international air travel restriction.

	Time elapsed from first world case to importation in Italy (in days) (95% CI)		
		Scenario	
**Level of air travel restriction**	Mild	Moderate	Severe
None	77 (55,92)	48 (34,57)	37 (25,42)
90%	94 (72,108)	59 (44,69)	44 (34,51)
99%	114 (92,127)	71 (55,79)	53 (42,60)

AVP appears to be the most effective single intervention, resulting in a 36%–76% reduction in cumulative ARs. It also contributes to delay the peak day (from 13 to 53 days) and to decrease the peak daily attack rate.

Vaccination impact strongly depends from its timing. In the mild scenario, it would reduce the cumulative AR by approximately 65%, if it is begun within 4 months of the first world case (Table 2). In the moderate and severe scenarios, ARs would be reduced by 42% and 31% respectively, if vaccination starts within 2 months and one month from the pandemic start (Table 2).

#### Combined measures

Table 4 shows the impact of combining vaccination with international air travel restrictions. In the mild scenario, there is no clear added value of air travel restriction. In the moderate and severe scenarios, the implementation of 99% of air travel restriction would allow to have one additional month to implement vaccination, since administering first dose within three months instead of two, for the moderate scenario, and within two months instead of one, for the severe scenario, would not modify cumulative Ars

**Table 4:** Clinical attack rates and peak day, combining vaccination with international air travel restriction, by scenario.

Control measure	Scenario								
	Mild			Moderate			Severe		
	Cumulative AR [%] (95% CI)	Peak day (95% CI)	Peak daily AR [%] (95% CI)	Cumulative AR [%] (95% CI)	Peak day (95% CI)	Peak daily AR [%] (95% CI)	Cumulative AR [%] (95% CI)	Peak day (95% CI)	Peak daily AR [%] (95% CI)
**90% air travel restriction, by vaccination timing from the first world case**									
1 month	7.3 (7.2–7.4)	277 (276–279)	0.07 (0.07–0.07)	17.8 (17.7–17.9)	162 (160–163)	0.37 (0.36–0.38)	27.1 (27.0–27.2)	106 (102–107)	0.96 (0.93–0.98)
2 months	7.3 (7.3–7.4)	277 (275–279)	0.07 (0.07–0.07)	17.8 (17.7–17.9)	155 (151–157)	0.38 (0.37–0.38)	34.1 (33.4–34.6)	97 (96–99)	1.91 (1.82–1.98)
3 months	7.4 (7.3–7.5)	275 (273–276)	0.07 (0.07–0.07)	20.2 (19.8–20.6)	129 (126,130)	0.54 (0.48–0.62)	38.7 (38.6–38.7)	98 (95–99)	1.93 (1.86–1.98)
4 months	7.3 (7.3–7.5)	265 (261–267)	0.07 (0.07–0.08)	29.6 (29.4–29.9)	139 (137–140)	1.04 (1.03–1.07)	38.7 (38.6–38.8)	98 (96–99)	1.93 (1.86–1.98)
**99% air travel restriction, by vaccination timing from the first world case**									
1 month	7.3 (7.2–7.4)	316 (311–324)	0.07 (0.07–0.07)	17.8 (17.7–17.9)	179 (177–180)	0.37 (0.36–0.37)	27.0 (27.0–27.1)	119 (118–121)	0.89 (0.79–0.95)
2 months	7.3 (7.3–7.4)	320 (313–324)	0.07 (0.07–0.07)	17.8 (17.7–17.9)	171 (167–175)	0.37 (0.35–0.38)	29.1 (28.6–29.4)	104 (101–109)	1.04 (0.99–1.11)
3 months	7.3 (7.2–7.4)	309 (302–315)	0.07 (0.07–0.08)	18.2 (18.1–18.4)	156 (152–161)	0.37 (0.36–0.38)	38.4 (38.3–38.5)	108 (105–109)	1.81 (1.59–1.94)
4 months	7.3 (7.2–7.4)	298 (291–305)	0.07 (0.06–0.07)	26.5 (25.9–26.8)	150 (148–153)	1.00 (0.95–1.03)	38.7 (38.6–38.8)	108 (104–109)	1.81 (1.59–1.94)

VE = 70%; vaccination target categories: personnel providing essential services, elderly persons, individuals 2–18 years of age

When combing all of the measures, in the mild scenario, the epidemic could be mitigated with moderate efforts. Specifically, performing vaccination for three target categories (i.e., personnel providing essential services, elderly persons, and 2–18 year-olds) and providing AVP to 90% of household contacts for the entire epidemic period would reduce the cumulative AR by 98% (from 21% to 0.3%), independently of the timing of vaccination (2, 3 or 4 months) and the implementation of air-travel restrictions. Limiting AVP to 8 weeks would produce a cumulative AR of 7.7%, which is similar to that observed with vaccination alone.

For the moderate scenario (Table 5, Figure 3), vaccinating the three above-mentioneded target categories and providing AVP to 90% of household contacts for the entire epidemic period, with 90% air travel restriction would reduce the cumulative AR by 77%–87% (from 31% to 4–7%, depending on the timing of the first vaccine dose). The cumulative AR is reduced by 87%, if 99% air-travel restrictions were implemented, and vaccination were begun within 4 months of pandemic start.

If air-travel restrictions were not implemented or were limited to the first two-months after the first national case, the AR decrease would be similar (81–87%), providing that vaccination were started within 3 months of the first world case. The cumulative AR would be even lower (2%, for first dose at 3 months) if AVP were provided to both household contacts and close contacts in schools and workplaces. This would require the administration of 11 millions of AV courses.

For the severe scenario (Table 6, Figure 3), the cumulative AR would decrease by 64% (from 39% to 14%) if the first vaccine dose were administered within 2 months of the first world case, AVP were provided to household contacts for the entire epidemic period, and 90% air-travel restriction were implemented, independently from its duration. The cumulative AR would further decrease (to 9%) if also vaccinating 40–64-year-old individuals, which would reduce the number of household contacts receiving AVP by 33%. If not vaccinating 40–64-year-olds and providing AVP to both household contacts and close contacts in schools and workplaces, the cumulative AR would decrease to 8%, though this would require an extremely high number of AVP doses (approximately 32 millions). Finally, with the implementation of 99% air-travel restriction, starting vaccination within three months of pandemic emergence would have the same impact than starting vaccination within two months, with no air-travel restrictions in place (cumulative AR = 16%). None of the other combinations of control measures would reduce the cumulative clinical AR to less than 16%.

Assuming a VE of 50% for all age-groups or a different VE by age group (i.e., 59% in individuals aged 2–18 years, 70% in individuals aged 40–64 years, and 40% in individuals ≥65 years) would not substantially affect the cumulative AR; in fact, the cumulative AR would be 2 or 3 percentage points higher, respectively, than observed assuming a 70% VE for all age groups (Tables 5 and 6).

**Table 5:** Clinical Attack rates and peak day, by combination of control measures.

Control measure	Cumulative AR [%] (95% CI)	Peak day (95% CI)	Peak daily AR [%] (95% CI)	Millions of AVP courses used	Millions of vaccine courses used
**90% air travel restriction**					
AVP°; fist vaccine dose at 2 months	4.5 (4.4–4.5)	213 (209–215)	0.04 (0.04–0.04)	6.8	14.4
AVP°; first vaccine dose at 3 months	4.6 (4.6–4.7)	186 (177–197)	0.04 (0.04–0.04)	7.1	14.4
AVP°; first vaccine dose at 4 months	6.7 (6.2–7.2)	154 (150–156)	0.15 (0.12–0.18)	10.0	14.4
AVP°; first vaccine dose at 2 months*	5.7 (5.6–5.7)	214 (206–219)	0.05 (0.05–0.06)	8.7	14.4
AVP°; first vaccine dose at 3 months*	5.8 (5.7–5.9)	194 (189–197)	0.06 (0.06–0.06)	8.9	14.4
AVP°; first vaccine dose at 4 months*	7.4 (7.2–7.8)	155 (151–156)	0.14 (0.12–0.16)	11.3	14.4
AVP°; first vaccine dose at 2 months†	7.1 (7.0–7.1)	211 (207–217)	0.07 (0.07–0.07)	10.8	14.4
AVP°; first vaccine dose at 3 months†	7.2 (7.1–7.3)	187 (179–194)	0.08 (0.08–0.08)	11.0	14.4
AVP°; first vaccine dose at 4 months†	8.6 (8.2–9.0)	155 (151–156)	0.15 (0.12–0.18)	13.1	14.4
AVP for 8 weeks; first vaccine dose at 3 months	18.2 (18.1–18.3)	171 (167–176)	0.38 (0.34–0.4)	0.1	14.4
AVP° plus school/workplace close contacts; first vaccine dose at 3 months	2.1 (2.0–2.2)	141 (127–166)	0.02 (0.02–0.03)	10.7	14.4
AVP°; first vaccine dose at 3 months, not vaccinating the elderly	5.3 (5.2–5.4)	192 (188–194)	0.05 (0.05–0.05)	8.0	8.5
AVP°; first vaccine dose at 3 months, vaccinating also adults	2.3 (2.2–2.3)	186 (184–188)	0.02 (0.02–0.02)	3.6	24.6
AVP°, first vaccine dose at 3 months, time-limited border restrictions**	5.1 (5.0–5.1)	165 (161–170)	0.05 (0.05–0.06)	7.8	14.4
**99% air travel restriction**					
AVP°; first vaccine dose at 2 months	4.4 (4.4–4.5)	274 (253–280)	0.04 (0.04–0.04)	6.7	14.4
AVP°; first vaccine dose at 3 months	4.4 (4.3–4.5)	251 (246–257)	0.04 (0.04–0.04)	6.7	14.4
AVP°; first vaccine dose at 4 months	4.6 (4.5–4.6)	222 (210–230)	0.04 (0.04–0.04)	7.0	14.4
**No air travel restriction**					
AVP°; first vaccine dose at 2 months	4.6 (4.6–4.8)	163 (162–165)	0.04 (0.04–0.04)	7.2	14.4
AVP°; first vaccine dose at 3 months	6.2 (6.1–6.3)	126 (123,129)	0.12 (0.12–0.13)	9.5	14.4
AVP°; first vaccine dose at 4 months	11.0 (10.8–11.1)	152 (151–156)	0.28 (0.27–0.28)	10.2	14.4

Moderate scenario (R_0_ = 1.7). VE = 70%; vaccination target categories: personnel providing essential services, elderly persons, indidividuals 2–18 years of age, unless otherwise specified.

AVP = antiviral prophylaxis;

* Different vaccine effectiveness for different categories: 59% in individuals 2–18 years of age [16], 70% in individuals 40–64 years of age [15], and 40% in ≥65 year-olds [17];

° Unlimited, household contacts;

† Vaccine effectiveness = 50%;

** air travel restrictions for 2 months after the first national case

**Table 6:** Clinical attack rates and peak day, by combination of control measures.

Control measure	Cumulative AR [%] (95% CI)	Peak day (95% CI)	Peak daily AR [%] (95% CI)	Millions of AVP courses used	Millions of vaccine courses used
**90% air travel restriction**					
AVP°; fist vaccine dose at 2 months	14.4 (14.4–14.5)	132 (130–134)	0.28 (0.28–0.29)	21.3	14.4
AVP°; first vaccine dose at 3 months	20.5 (20.1–20.8)	124 (122–125)	0.79 (0.77–0.80)	29.1	14.4
AVP°; first vaccine dose at 4 months	24.6 (24.5–24.7)	124 (122–125)	0.79 (0.77–0.80)	34.9	14.4
AVP°; first vaccine dose at 2 months*	16.2 (16.1–16.3)	130 (129–131)	0.34 (0.33–0.34)	23.7	14.4
AVP°; first vaccine dose at 3 months*	21.1 (21.0–21.3)	124 (123–127)	0.78 (0.77–0.80)	30.0	14.4
AVP°; first vaccine dose at 4 months*	24.7 (24.6–24.7)	124 (122–126)	0.78 (0.77–0.80)	35.0	14.4
AVP°; first vaccine dose at 2 months†	17.6 (17.4–17.7)	130 (127–132)	0.40 (0.39–0.40)	25.8	14.4
AVP°; first vaccine dose at 3 months†	21.6 (21.3–22.0)	124 (122–125)	0.79 (0.77–0.80)	30.8	14.4
AVP°; first vaccine dose at 4 months†	24.7 (24.6–24.8)	124 (122–125)	0.79 (0.77–0.80)	35.0	14.4
AVP for 8 weeks; first vaccine dose at 2 months	27.4 (27.3–27.4)	126 (123–130)	0.98 (0.97–0.99)	1.5	14.4
AVP° plus school/workplace close contacts; first vaccine dose at 2 months	7.9 (7.7–8.1)	117 (101–127)	0.14 (0.13–0.15)	31.8	14.4
AVP°; first vaccine dose at 2 months, not vaccinating the elderly	16.0 (16.0–16.1)	131 (129–134)	0.32 (0.31–0.33)	23.2	8.5
AVP°; first vaccine dose at 2 months, vaccinating also adults	9.0 (8.8–9.3)	126 (119–132)	0.16 (0.15–0.17)	13.6	24.6
AVP°, first vaccine dose at 2 months, time–limited air travel restrictions**	14.7 (14.6,14.8)	125 (121–128)	0.29 (0.28–0.3)	21.8	14.4
**99% air travel restriction**					
AVP°; fist vaccine dose at 2 months	14.2 (14.1–14.3)	156 (152–158)	0.27 (0.26–0.28)	21.0	14.4
AVP°; first vaccine dose at 3 months	15.8 (15.5–16.3)	129 (127,131)	0.39 (0.35–0.47)	22.9	14.4
AVP°; first vaccine dose at 4 months	23.3 (23.0–23.6)	139 (137–141)	0.77 (0.75–0.79)	33.0	14.4
**No air travel restriction**					
AVP°; fist vaccine dose at 2 months	16.5 (16.1–16.7)	99 (97–101)	0.49 (0.43–0.52)	24.0	14.4
AVP°; first vaccine dose at 3 months	23.7 (23.4–23.8)	109 (107–111)	0.75 (0.75–0.76)	33.6	14.4
AVP°; first vaccine dose at 4 months	24.8 (24.7–24.9)	109 (107–111)	0.75 (0.75–0.76)	35.3	14.4

Severe scenario (R_0_ = 2). VE = 70%; vaccination target categories: personnel providing essential services, elderly persons, individuals 2–18 years of age, unless otherwise specified.

AVP = antiviral prophylaxis;

* Different vaccine effectiveness for different categories: 59% in individuals 2–18 years of age [16], 70% in individuals 40–64 years of age [15], and 40% in ≥65 year-olds [17];

° Unlimited, household contacts;

† Vaccine effectiveness = 50%;

** air travel restrictions for 2 months after the first national case

**Figure 4:**
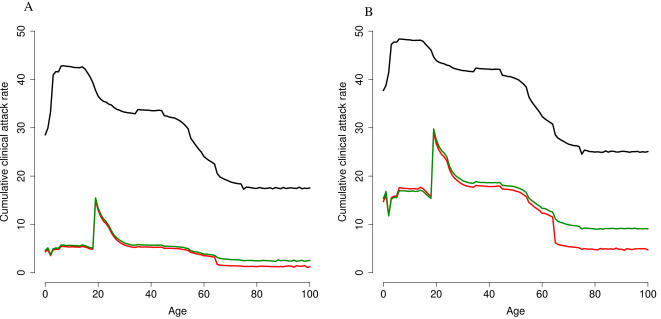
Cumulative clinical AR, by age and scenario (Panel A: R_0_ = 1.7; Panel B: R_0_ = 2). Black line represents baseline results; red line represents the standard vaccination strategy (i.e., personnel providing essential services; elderly persons; and 2–18 year-olds); green line represents the effect of limiting vaccination to essential workers and children.

Figure 4 shows the cumulative AR by age and vaccination strategy. If no control measures were performed (baseline), the cumulative AR would be highest for individuals ≤18 years of age and would decrease with increasing age. None of the considered scenarios included vaccinating 18–25-year-old individuals, who consequently appear to be the age-group with the highest incidence after vaccination. However, if vaccinating personnel providing essential services (15% of the 25–60-year-old working population), elderly persons (≥65-year-olds), and 2–18 year-olds, the AR would also decrease among individuals 19–64 years of age, who are not targeted by vaccination. In particular, the AR would decrease by approximately 75% in unvaccinated 30–50-year-old individuals. Excluding the elderly from vaccination would not affect the cumulative AR in the other age groups.

### Conclusions

Recent modelling studies have estimated that the first cases of influenza in a future pandemic would be imported to Europe within 50–90 days of its emergence elsewhere in the world [9], [18]. Our results indicate that the first cases would be imported to Italy within 37–77 days, depending on the R_0_, and that the incidence would peak 54–125 days after importation. When considering separately the three scenarios in our study, the timing of the peak for the severe scenario (i.e., 54 days) was similar to that for the severe scenario in the UK (i.e., 50 days), whereas it differed for the moderate scenario (i.e., 77 days for Italy compared to 65 days for the UK) [9]. The reason for this divergence is likely due to the different R_0 _values considered in the global SEIR model, which were scaled in order to be proportional to those considered in the national IBM simulations (i.e., 1.4, 1.7, and 2). Varying the global R_0_, can in fact substantially modify the timing of national first case introduction, and the consequent epidemic peak. The lower number of air travellers coming into Italy per year compared to US and UK (25 millions, versus 73 and 92 millions, respectively) [9], could also play a role in explaining this difference.

It is widely accepted that a combination of measures would be necessary to sufficiently control the spread of an influenza pandemic, specifically, vaccination, AVP, social distancing, and air travel restrictions [9]–[11]. In our simulations, AVP is confirmed to be the most effective single intervention [11]; however this would require to stockpile a high number of antivirals, to be capable to rapidly identify index cases, to treat a high number of contacts, and to maintain their compliance to a treatment lasting 10 days.

Recent modelling studies have predicted that the use of a pre-pandemic vaccine with a low VE after the first dose (i.e., 30%) would be crucial for pandemic mitigation if the R_0_ were 1.7 yet not higher [10]. In our model, we introduced pre-pandemic vaccine for population priming and considered the vaccine to be effective only after the administration of a successive dose of pandemic vaccine, assuming different hypotheses for VE. In particular, we were interested in determining whether variations in VE by age could provide further insight into the impact of control measures. Systematic reviews have shown that the clinical effectiveness of seasonal influenza vaccine varies with age, with a higher VE in adults than in children and the elderly (70% vs. 59% and 40%, respectively) [15], [17], [27]. Our results show that these differences would not substantially affect the cumulative AR. Moreover, vaccinating 2–18 year-olds would reduce by approximately 75% the AR in unvaccinated 30–50-year-old adults, showing a clear herd immunity effect. These results thus support the idea that, during a pandemic, vaccinating children should be a higher priority than vaccinating elderly persons [11], [28].

With specific regard to air-travel restrictions, the effectiveness of this measure remains controversial [9], [13], [14], [29]–[33]. Our results confirm that international air-travel restrictions can buy about 1 to 3 weeks in delaying the epidemic [9], [14], [32], [33]. In the moderate and severe scenarios, the implementation of 99% air-travel restriction, would allow to gather one-two months of time for administering the vaccine to target population. In detail, if R_0_ were 2, starting vaccination within three months of pandemic emergence would have the same impact than starting vaccination within two months of the first world case, with no air-travel restrictions in place.

However, the administration of the first vaccine dose within three months of the first world case would be possible only if vaccines against “high pandemic risk” avian influenza strains (such as A/H5N1) were stockpiled before the pandemic. In any case, because of the antigenic drift of the virus, it is not possible to precisely predict the effectiveness of pre-pandemic vaccines. In this scenario, it is reassuring that a decrease in VE from 70% to 50% would not significantly modify the impact of vaccination.

When using a pre-pandemic vaccine, the maximum reduction in the AR would be achieved by either providing AVP to both household contacts and close contacts in the school/workplace, as shown in a previous work [11] (i.e., 32 million antiviral courses, covering approximately 56% of the national population), or by vaccinating adults (i.e., 25 million vaccine courses), in addition to the other target categories. In the occurrence of an actual pandemic, the choice of the strategy will be based on several factors which at present are unknown, such as the capacity to produce vaccines, the effectiveness of vaccination and AVP, and logistic constraints in the distribution of vaccines and AVP.

In interpreting the results of this model, some limitations need to be mentioned. The model requires detailed information on the population's characteristics, including age and geographic distribution, the size of households, schools and workplaces, and commuting data. In our study, the source of these data were routinely collected national statistics. The number of students per school and workers per workplace vary in proportion to the resident population in the different geographic areas. However, we assumed that the employment rate was the same throughout Italy, though it is known to vary greatly when comparing northern, central, and southern Italy (4%, 6% and 12%, respectively) [34]. Moreover, in modelling the social distancing measures, we only considered the closing of those public workplaces not providing essential services, which could have resulted in an underestimate of the effect of such measures. Furthermore, these workplaces are probably not uniformly distributed throughout Italy.

In the global SEIR model we considered all infected persons to be symptomatic and not travelling; thus we may have overestimated the effect of travel restrictions. By contrast, national data on in-coming flow by land and sea were not easily available, and we therefore did not take into account land and sea importation and control. This could also have overestimated the effect of travel restrictions, since importation via all routes should be considered and eventually reduced. Furthermore, a number of factors, which we did not consider in our analysis, could modify the effects of the delay caused by air-travel restrictions, in particular, seasonality [32], environmental effects, and viral evolution, whereas we assumed that contact, transmission and disease parameters remained constant throughout the pandemic period in Italy. Also, we did not include disease-related mortality, considering that deaths would probably occur at the latter stages of the infectious period and thus would not affect the diffusion of disease.

Despite these limitations, and considering that we cannot predict all aspects of an actual pandemic, this IBM, which is based on country-specific demographic data, could be suitable for the real-time evaluation of measures to be undertaken in the event of the emergence of a new pandemic influenza virus.

## Supporting Information

Text S1 Supporting information contains details on the model: socio-demographic and commuting structure, and transmission model.
(0.15 MB PDF)

Table S1 Percentage of different household types. * with additional household member.
(0.01 MB PDF)

Table S2 Household size by type (in percentage). *with additional household member.
(0.01 MB PDF)

Table S3 Age class of household heads in couples with children by household size (in percentage).
(0.01 MB PDF)

Table S4 Age class of household head in couples without children.
(0.01 MB PDF)

Table S5 Age class of singles without children.
(0.01 MB PDF)

Figure S1 (0.30 MB TIF)

Figure S2 (1.56 MB TIF)

Figure S3 (1.05 MB TIF)

Figure S4 (0.38 MB TIF)

Figure S5 (0.98 MB TIF)

Figure S6 (0.51 MB TIF)

Figure S7 (0.62 MB TIF)

Figure S8 (0.93 MB TIF)

Figure S9 (0.72 MB TIF)

Figure S10 (0.30 MB TIF)
