# Supplementary material for: Mitigation Measures for Pandemic Influenza in Italy: An Individual Based Model Considering Different Scenarios
Source: PLoS One. 2008 Mar 12;3(3):e1790. doi: 10.1371/journal.pone.0001790 (PMC2258437; doi:10.1371/journal.pone.0001790)
Supplement: Text S1 — Supporting information contains details on the model: socio-demographic and commuting structure, and transmission model. (0.15 MB PDF) [file pone.0001790.s001.pdf]

Mitigation measures for pandemic influenza in Italy: an individual based model considering different scenarios

Supporting information

Marta Luisa Ciofi degli Atti[1], Stefano Merler[2], Caterina Rizzo[1,3] , Marco Ajelli[2,4], Marco Massari[1], Piero Manfredi[5], Cesare Furlanello[2], Gianpaolo Scalia Tomba[6],Mimmo Iannelli [7]

Contents

1 Introduction **1**

2 Sociodemographic model **2**
2.1 Population data 2
2.2 Households 2
2.3 Employment 2
2.4 Commuting 3

3 Epidemiological model **4**
3.1 The individual based model 4
3.2 Estimating R_0 6
3.3 Number of imported cases 7
3.4 Comparison with past pandemics 7

4 Software **8**

References **9**

1 Introduction

The model, adapted from that proposed by [4, 5], is a stochastic, spatially structured, individual based model with discrete time simulation. Infection spreads within households, within schools and workplaces, by random contacts in the population (see also [6, 8, 7]). The models consists of (A) a sociodemographic model, in which individuals are co-located in households, schools, workplaces on the basis of census and commuting data, (B) a local epidemiological model describing the temporal evolution of the flu epidemic in the country considered (Italy)

[1]National Center for Epidemiology Surveillance and Health Promotion, Istituto Superiore di Sanità, Rome, Italy
[2]Fondazione Bruno Kessler, Trento, Italy
[3]Department of Pharmaco–Biology, University of Bari, Italy
[4]Information Engineering and Computer Science Department, University of Trento, Italy
[5]Department of Statistics and Mathematics Applied to Economics, University of Pisa, Italy
[6]Department of Mathematics, University of 'Tor Vergata', Roma, Italy
[7]Department of Mathematics, University of Trento, Italy

and (C) a global SEIR model describing the worldwide temporal evolution of the flu epidemic and allowing for the estimate of the number of imported cases by international flights.

2 Sociodemographic model

2.1 Population data

Population data of Italy (56,995,744 individuals) are obtained from census data [10] (382,534 census sections) and are hierarchically grouped by municipalities (8,101), provinces (103) and regions (20), according to the administrative borders of the study area (see Fig. S1a). Number of individuals by municipality ranges from 33 to 2,546,804 (Rome municipality) with an average of 7,035 and a standard deviation of 39,326 (see Fig. S2a). Only 42 municipalities contain more than 100,000 individuals (6 of which contain more than 500,000), while 1,971 contain less than 1,000 individuals (37 of which less than 100). By approximating the districts as circles, the mean radius of the municipalities is 3.02 km (SD=1.64 km), the mean radius of the provinces is 13.01 km (SD=3.56 km), the mean radius of the regions is 67.01 km (SD=18.32 km).

2.2 Households

Census data on age structure and household type and size [11] are used to randomly assign age and co-locate individuals in households. These data refer to the analysis of 19,227 households, corresponding to approximately 0.1% of the Italian households. Nine different types of household are considered: they are described in Table S1. Their size is reported in Table S2. The age of the household head for some of the different household types considered is reported in Table S3 (couples with children), Table S4 (couples without children) and Table S5 (singles without children). Note that the description "without children"applies either to families without children or with children which do not live with the parents anymore. The following additional constraints are also considered when assigning individuals to households:

C1 any household must contain at least 1 adult (age $\geq$ 18);

C2 the age of any child is between 43 and 18 years less than that of the youngest parent;

C3 spouses age differs by no more than 15 years.

The algorithm employed for generating individuals, assigning age an co-locating individuals in households is described in Fig. S3. A comparison between real and simulated age structure is reported in Fig. S2b. A comparison between real and simulated data on household size is reported in Fig. S2c (note that these data are not directly employed in the algorithm developed for generating individuals and co-locating them in households).

2.3 Employment

Demographic, school [12, 13] and industry [9] census data are used for randomly assigning an employment category to each individual on the basis of age. The Italian population at 2001

is structured as follows: 20,559,595 workers, 11,360,556 students and 25,084,274 unemployed or retired. Students are deterministically assigned to a specific school type (6 types, from nursery school to university) on the basis of age. Workers are assigned to a random workplace type (7 types, depending on the workplace size, i.e., the number of employees, see Fig. S2d). Students are grouped in classes, whose average size depends on the type. Specifically, the average size has been set to 20 in nursery schools, 40 in kindergartens, 19 in primary school, 21 in middle and high schools, 34 in universities [12, 13]; we also consider close colleagues of workers: in small workplaces (less than 10 employees) all workers are close colleagues. In bigger workplaces employees are clustered in groups of size 5.

2.4 Commuting

Commuting destination are assigned in order to fit available commuting data [10]. In particular, the proportion of individuals with age ≥ 15 working or attending school in the same municipality of residence (N) is available for each municipality, together with the number of individuals traveling either to a municipality of the same province they live in (P), or outside the province but within the same region (R), or outside the region (S).

As a starting point, for determining the probability of commuting from municipality i to municipality j we employed a gravity model of the form [14]

$$C_{ij} = \theta \frac{p_i^{\tau_i} p_j^{\tau_j}}{d_{ij}^{\rho}} \ , \tag{1}$$

where p_i and p_j are the number of individuals living in municipality i and j respectively and d_{ij} is the distance between the two municipalities. θ is a proportionality constant, τ_i and τ_j tune the dependence of dispersal on donor and recipient sizes and ρ tunes the dependence on the distance.

The proportion of commuters (individuals traveling outside the municipality of residence for work or school) in Italy, however, varies significantly by province. In particular, the proportion of commuters drastically increases from South to North of Italy (see Fig. S4). The proportion of commuters varies from 15% to 60% and this variability does not depend on the size of the municipalities or on the distance among municipalities. Indeed, it depends on social factors and thus can not be explained by model (1). Thus, we considered model (1) with an additional constraint forcing the model to produce in each province the proportion of commuters as resulting from the available data.

The set of model parameters was optimized by searching for the set giving rise to a simulated population of commuters matching the available data on the number of individuals commuting within the province, the region or outside the region of residence. We obtained the following estimates: $\tau_i = 0.28$, $\tau_j = 0.66$ and $\rho = 2.95$ which are very close to those obtained in [14] for modeling travel destinations in the US at short distances (less than 119 km). Fig. S5a-c shows how well the model fit the available commuting data. Fig. S5d shows the resulting distance to work/school distribution. Schools and workplaces are finally generated to fit data on number and size and commuting data.

3 Epidemiological model

3.1 The individual based model

For each individual i we define:

- H_i as the set of the n_i individuals belonging to the same household of the individual i;
- L_i^j as the set of the m_i^j individuals attending the same school (index $j = 1, \ldots, 6$ identifies school type) or sharing the same workplace (index $j = 7, \ldots, 13$ identifies workplace type) of the individual i;

Any susceptible individual i, at any time t of the simulation has a probability $p_i = 1 - e^{-\lambda_i \Delta t}$ of becoming infected, where $\Delta t = 0.25$ days is the time-step of the simulation and λ_i is the instantaneous risk of infection. The latter is the sum of the risks coming from the three source of infections:

1. contacts with infectious members of the household (first term in Eq. 2),
2. contacts with infectious individuals working in the same workplace or attending the same school (second term in Eq. 2),
3. random contacts in the population (third term in Eq. 2).

$$\begin{aligned}
\lambda_i &= \sum_{k|H_k=H_i} \frac{I_k \beta_h \kappa(t-\tau_k)\left[1 + C_k(\omega - 1)\right]}{n_i^\alpha} \\
&+ \sum_{j,k|L_k^j=L_i^j} \frac{I_k[1 + A_k(\xi - 1)]\beta_p^j \kappa(t-\tau_k)\left[1 + C_k(\omega \psi_p^j(t-\tau_k) - 1)\right]}{m_i^j - c_i^j} \\
&+ \sum_{k=1}^{N} \frac{I_k \beta_r \kappa(t-\tau_k) f(d_{ik})\left[1 + C_k(\omega - 1)\right]}{\sum_{k=1}^{N} f(d_{ik})} \qquad (2)
\end{aligned}$$

The terms in Eq. (2) are defined as follows:

- N is the total population;
- $I_k = 1$ if individual k is infected, 0 otherwise;
- β_h/day is the within–household transmission coefficient, β_p^j are the within–school/workplace transmission coefficients and β_r is the transmission coefficient for random contacts.
- τ_i is the time individual i became infectious and $\kappa(T)$ is a lognormal function describing infectiousness over time. Estimates of incubation period (1.48 ± 0.47 days) and infectiousness period ($\int_0^\infty T\kappa(T)dT$) lead to a generation time $T_g = 2.6$ days (as in [4]);
- $C_k = 1$ for symptomatic cases (we suppose the 50% of cases to be symptomatic), 0 otherwise. Since $\omega = 2$, the infectiousness of symptomatic cases doubles the one of asymptomatic cases (as in [4]);

- $\alpha = 0.8$ scales the household transmission rates with household size (as in [4]);
- $\psi_p^j(T)$ is a function accounting for induced absenteeism and it is defined as follows: if $T > 0.25$ (the minimum time for recognizing the infection) $\psi_p^j(T)$ is set to 0.1 for $j = 1, 2$, 0.2 for $j = 3, 4$, 0.25 for $j = 5$ and 0.5 for $j = 6, \ldots, 13$, 1 otherwise;
- $A_k = 1$ if individual k is a close contact of individual i, 0 otherwise. The parameter $\xi > 1$ is used to weight the transmission of close contacts with respect to generic contacts. c_i^j is the number of close contacts of individual i (see Tab. 1 in the main text).

The second term of Eq. (2) accounts for the transmission of infection in schools and workplaces due to both generic and close contacts. Let β_{pc}^j be the transmission coefficient among close contacts. In order to make the contribution to the force of infection of the c_i^j close contacts ξ times that of the $m_i^j - c_i^j$ generic contacts, the following relation must hold:

$$\frac{\beta_{pc}^j}{c_i^j} = \xi \frac{\beta_p^j}{m_i^j - c_i^j} \ .$$

It follows that $\beta_{pc}^j = \xi \frac{c_i^j}{m_i^j - c_i^j} \beta_p^j$. Consequently, the contribution to the force of infection in schools and workplaces due to both generic and close contacts is given by

$$\begin{aligned} & \sum_{j,k | F_k^j = F_i^j} \frac{I_k \xi \frac{c_i^j}{m_i^j - c_i^j} \beta_p^j \kappa(t - \tau_k) \left[1 + C_k(\omega \psi_p^j(t - \tau_k) - 1)\right]}{c_i^j} \\ + & \sum_{j,k | G_k^j = G_i^j} \frac{I_k \beta_p^j \kappa(t - \tau_k) \left[1 + C_k(\omega \psi_p^j(t - \tau_k) - 1)\right]}{m_i^j - c_i^j} \\ = & \sum_{j,k | L_k^j = L_i^j} \frac{I_k [1 + A_k(\xi - 1)] \beta_p^j \kappa(t - \tau_k) \left[1 + C_k(\omega \psi_p^j(t - \tau_k) - 1)\right]}{m_i^j - c_i^j} \end{aligned}$$

where

- F_i^j is the set of the c_i^j close contacts of the individual i attending the same school/workplace;
- $G_i^j = L_i^j \setminus F_i^j$ is the set of the $m_i^j - c_i^j$ generic contacts of the individual i attending the same school/workplace.

We set $\xi = 2$.

The third term of Eq. (2) accounts for the transmission due to random contacts with infectious individuals in the population. As in [4, 5], we assume that an infectious individual k can infect any susceptible individual i in the population and we assume that transmission depends explicitly on the distance between infectious individual k and susceptible individual i. The probability that an infectious individual k infects individual i is weighted by the kernel function

$$f(d_{ik}) = \frac{1}{1 + (d_{ik}/a)^b} \tag{3}$$

which is a decreasing function of the distance d_{ik} between the two individuals. Parameters a and b where optimized by employing Eq. (3) for generating a synthetic population of commuters such that the resulting distance to work/school distribution matches that obtained by employing the gravity model (1) (see Fig. S5d). The estimated parameters are $a = 3.8km$ and $b = 2.32$.

3.2 Estimating R_0

A practical method for computing the basic reproductive number of an IBM is to consider the first generation index G_0, which is the average number of secondary infections generated by the first infectious individual and to approximate R_0 by averaging G_0 over many simulations. However, the resulting estimate of R_0 is systematically lower than that obtained by employing Eq. (4) or (5) and the difference increases with R_0 [6, 5, 2].

Another problem is that the infectiousness in the IBM is not constant over time and thus classical methods for estimating R_0 for SEIR models [3],

$$R_0 = 1 + \frac{r^2 + r(\omega + \gamma)}{\omega\gamma} \ , \tag{4}$$

or SIR models

$$R_0 = 1 + \frac{r}{\gamma} \ , \tag{5}$$

where $1/\omega$ is the the incubation period, $1/\gamma$ is the infectious period and r is the initial per-capita rate of growth that can be estimated as the regression coefficient of a linear model fitted to the logarithm of the cumulative number of cases $y(t)$, that is by assuming $y(t) \approx e^{rt}$, are not suitable.

In [5], a method for computing the reproductive number for discrete-time simulations with infectiousness not constant over time has been proposed:

$$R_0 = \frac{1}{e^{-r\Delta t} \sum_{m=0}^{\infty} \kappa(m\Delta t) e^{-rm\Delta t}} \tag{6}$$

where $\kappa(T)$ is the infectiousness T days after infection, normalized to be a probability density function and Δt is the time step of the simulation.

For estimating the basic reproductive number R_0 of the simulation model we employed Eq. (6). More in detail, we initialized simulations with 100 infected individuals at time 0 (and no imported cases) and estimated the rate of growth of the cumulative number of cases when it stabilizes (see Fig. S6). R_0 where estimated by applying Eq. (6) and by averaging over many simulations.

As in [5], we found that a good approximation (with R_0 approximately proportional to the real time growth rate r) of Eq. (6) is given by

$$R_0 = 1 + rT_e \ , \tag{7}$$

with $T_e = 3.2$ days that is slightly larger than the generation time of the model $T_g = 2.6$ days, though the estimates tend to differ in the severe scenario.

3.3 Number of imported cases

To estimate the number of imported cases by international flights we employed different global deterministic SEIR models (depending on the basic reproductive number R_0) describing the worldwide temporal evolution of the epidemics.

More in detail, the number of imported cases at each step of the simulation is obtained by sampling a Poisson distribution with parameter $\Delta t E(t) a/N$, where $E(t)$ is the number of exposed individuals obtained with the SEIR model at time t, N is the world population, $a \approx 70,000$/day is the total number of persons arriving daily in Italy and Δt is the time step of the simulation (see Fig. S7).

To parametrize the global SEIR models, we fixed the latent period to $1/\omega = 1.5$ days (as in the IBM) and than we set the infectious period to $1/\gamma = 1.5$ days in order to give approximatively the same real time growth rate as in the IBM, namely r =0.12/day, r =0.21/day and r =0.3/day for $R_0 = 1.4$, $R_0 = 1.7$ and $R_0 = 2$ respectively.

By assuming that only 50% of cases are symptomatic, the cumulative clinical attack rate of the SEIR model for $R_0 = 2$ is 39.8%, the peak day is at 80 days and the clinical peak attack rate is 2.6%. For $R_0 = 1.7$, the cumulative clinical attack rate is 34.5% with peak day at 106 days and clinical peak attack rate 1.7%. For $R_0 = 1.4$, the cumulative clinical attack rate is 25.5%, with peak day at 170 days and clinical peak attack rate 0.7%.

This choice does not account for possible international travel restriction measures and of course results can change drastically by changing the length of the infectious period.

In [5], a unique SEIR model was employed for seeding the simulations of all scenarios. The R_0 was set to 1.6 with latent period of $1/\omega$ =1.5 days and infectious period adjusted to give a real time growth rate of r =0.2/day (as in the IBM). Also, in our model we import a lower number of cases since the number of travelers is 25 millions for Italy, 92 millions for GB and 73 millions for US, even though we employ a global SEIR model with the same R_0 as in the IBM.

Figure S8 shows the stochastic variability in timing of initial case in the baseline scenario and when travel restrictions measures are considered.

As regards the seeding municipalities, we decided to locate infected individuals arriving daily in the Italian international airports in the municipalities hosting the airports themselves. In fact, detailed information on the final destinations of travelers or spatial data on the number of tourists are not available to us. The choice adopted in [5] for GB and UK to locate travelers proportionally to population density is not suitable for Italy since sea and mountain touristic localities are, in general, low population municipalities. Moreover, data on travelers by sea and land were not available to us and were not considered in the model.

3.4 Comparison with past pandemics

Since daily or weekly historical data on past pandemics in Italy are not available, we compared simulation results with data on weekly deaths in GB during the first two waves of the 1918-19 Spanish pandemic. Of course, the results of this comparison have to be considered cautiously. In fact, the sociodemography of today Italy and 1918-19 GB are quite different, especially as regards the size of households. Moreover, while we considered our baseline model for this comparison, individual behavior could have been modified during the curse of the pandemic

(for instance, workplaces and school closure could have be considered when incidence exceeded a certain threshold). However this is the same procedure considered in [5]. First of all, by assuming an incubation period $1/\omega$ of 1.5 days, we employed Eq. (4) (with $1/\gamma = 1.5$ days as in the global SEIR model for cases importation), Eq. (5) (with $1/\gamma = T_g = 2.6$ days) and Eq. (7) for estimating R_0 during the first two waves of the 1918-19 pandemic in GB.

For the first peak we obtained estimates of R_0=2.1, R_0=1.9 and R_0=1.8 for the three methods respectively. For the second peak we obtained R_0=1.7, R_0=1.6 and R_0=1.5. The estimates comply with those obtained in [5] where the last two methods were employed. Fig. S9 shows the comparison between the observed data and the output of two baseline simulations with $R_0 = 1.9$ and $R_0 = 1.6$ for the first and second wave respectively. The poor fit observed in the decay phase of the second wave my be due to military demobilization at the end of the first world war. Fig. S10 compares clinical attack rates by age for a simulation with $R_0 = 1.6$ with the data reported in [7] on the 1918-19 pandemic. These results also support our choice of incubation period and infectious period for parameterizing the individual based model.

4 Software

The software implementing the algorithm described in this work is available at the URL `http://mpa.fbk.eu/static/merler/ibmflu`. It is available as a gzipped archive containing the source code in C. It contains a README file with information for compiling it (under Unix/Linux machines) and a short description of program parameters, input and output files. It requires GNU Scientific Library (`http://www.gnu.org/software/gsl`) and Sqlite (`http://www.sqlite.org/`).

References

[1] School of Population Health, University of Melbourne, Australia. FluWeb Influenza Historical Resources Database. Availibale on-line at `http://influenza.sph.unimelb.edu.au/`.

[2] M. Ajelli and S. Merler. The impact of the unstructured contacts component in influenza pandemic modeling. *PLoS ONE*, 3(1):e1519, 2008.

[3] G. Chowell, N. W. Hengartner, C. Castillo-Chavez, P. W. Fenimore, and J. M. Hyman. The basic reproductive number of ebola and the effects of public health measures: the cases of congo and uganda. *J Theor Biol*, 229(1):119–126, Jul 2004.

[4] N. M. Ferguson, D. A. Cummings, S. Cauchemez, C. Fraser, S. Riley, A. Meeyai, S. Iamsirithaworn, and D. S. Burke. Strategies for containing an emerging influenza pandemic in Southeast Asia. *Nature*, 437:209–214, 2005.

[5] N. M. Ferguson, D. A. Cummings, C. Fraser, J. C. Cajka, P. C. Cooley, and D. S. Burke. Strategies for mitigating an influenza pandemic. *Nature*, 2006.

[6] T. C. Germann, K. Kadau, I. M. Jr Longini, and C. A. Macken. Mitigation strategies for pandemic influenza in the United States. *Proc Natl Acad Sci USA*, 103(15):5935–4, April 2006.

[7] W. P. Glezen. Emerging infections: pandemic influenza. *Epidemiol Rev*, 18(1):64–76, 1996.

[8] I. M. Jr Longini, A Nizam, S Xu, K Ungchusak, W Hanshaoworakul, D. A. Cummings, and M. E. Halloran. Containing pandemic influenza at the source. *Science*, 309(5737):1083–7, August 2005.

[9] Italian Institute of Statistics (ISTAT). VIII Censimento generale dell'industria e dei servizi . Availibale on-line at `http://dwcis.istat.it/cis/index.htm`, 2001.

[10] Italian Institute of Statistics (ISTAT). XIV Censimento generale della popolazione e delle abitazioni. Availibale on-line at `http://dawinci.istat.it/MD/`, 2001.

[11] Italian Institute of Statistics (ISTAT). Strutture familiari e opinioni su famiglia e figli. Availibale on-line at `http://www.istat.it/dati/catalogo/20060621_03/`, 2003.

[12] Italian Ministry of University and Reasearch (MIUR). La scuola in cifre. Availibale on-line at `http://www.miur.it/ustat/documenti/pub2005/`, 2005.

[13] Italian Ministry of University and Reasearch (MIUR). L'università in cifre. Availibale on-line at `http://www.miur.it/ustat/documenti/pub2005/`, 2005.

[14] C. Viboud, O.N. Bjrnstad, D.L. Smith, L. Simonsen, M.A. Miller, and B.T. Grenfell. Synchrony, waves, and spatial hierarchies in the spread of influenza. *Science*, 312(5772):447–451, 2006.
