# Supplementary material for: Mitigation Measures for Pandemic Influenza in Italy: An Individual Based Model Considering Different Scenarios
Source: PLoS One. 2008 Mar 12;3(3):e1790. doi: 10.1371/journal.pone.0001790 (PMC2258437; doi:10.1371/journal.pone.0001790)
Supplement: Table S1 — Percentage of different household types. * with additional household member. (0.01 MB PDF) [file pone.0001790.s002.pdf]

Table S1: *Percentage of different household types.* * *with additional household member.*

type	percentage
single without children	25.8
single with children	7.9
single with children*	0.6
couple without children	19.7
couple without children*	1.3
couple with children	40
couple with children*	1.9
adults living together	1.6
more household groups	1.2
