# Supplementary material for: Mitigation Measures for Pandemic Influenza in Italy: An Individual Based Model Considering Different Scenarios
Source: PLoS One. 2008 Mar 12;3(3):e1790. doi: 10.1371/journal.pone.0001790 (PMC2258437; doi:10.1371/journal.pone.0001790)
Supplement: Table S2 — Household size by type (in percentage). *with additional household member. (0.01 MB PDF) [file pone.0001790.s003.pdf]

Table S2: *Household size by type (in percentage).* * *with additional household member.*

type	household size					
	1	2	3	4	5	≥ 6
single without children	100	0	0	0	0	0
single with children	0	68.9	25	6.1	0	0
single with children*	0	0	68.9	25	6.1	0
couple without children	0	100	0	0	0	0
couple without children*	0	0	100	0	0	0
couple with children	0	0	45.1	43.8	9.5	1.6
couple with children*	0	0	0	45.1	43.8	11.1
adults living together	0	78.5	21.5	0	0	0
more household groups	0	0	0	49.3	44.3	6.4
