# Supplementary material for: Mitigation Measures for Pandemic Influenza in Italy: An Individual Based Model Considering Different Scenarios
Source: PLoS One. 2008 Mar 12;3(3):e1790. doi: 10.1371/journal.pone.0001790 (PMC2258437; doi:10.1371/journal.pone.0001790)
Supplement: Table S3 — Age class of household heads in couples with children by household size (in percentage). (0.01 MB PDF) [file pone.0001790.s004.pdf]

Table S3: *Age class of household heads in couples with children by household size (in percentage).*

household size	age class						
	18–24	25–34	35–44	45–54	55–64	65–74	≥ 75
3	1.7	18.8	23.3	23.7	21.4	9.1	2
4	0.5	14.4	43.6	28.8	10.7	1.8	0.2
≥ 5	0.3	9.8	45.5	33.7	8.7	1.7	0.2
