# Supplementary material for: Mitigation Measures for Pandemic Influenza in Italy: An Individual Based Model Considering Different Scenarios
Source: PLoS One. 2008 Mar 12;3(3):e1790. doi: 10.1371/journal.pone.0001790 (PMC2258437; doi:10.1371/journal.pone.0001790)
Supplement: Table S4 — Age class of household head in couples without children. (0.01 MB PDF) [file pone.0001790.s005.pdf]

Table S4: *Age class of household head in couples without children.*

age class	percentage
15–34	8
35–44	17.8
45–54	22.1
$\geq$ 55	52.1
