# Supplementary material for: Mitigation Measures for Pandemic Influenza in Italy: An Individual Based Model Considering Different Scenarios
Source: PLoS One. 2008 Mar 12;3(3):e1790. doi: 10.1371/journal.pone.0001790 (PMC2258437; doi:10.1371/journal.pone.0001790)
Supplement: Table S5 — Age class of singles without children. (0.01 MB PDF) [file pone.0001790.s006.pdf]

Table S5: *Age class of singles without children.*

age class	percentage
15–24	1.5
25–44	24.2
45–64	21.8
≥ 65	52.5
