# Supplementary figures and images for: Mitigation Measures for Pandemic Influenza in Italy: An Individual Based Model Considering Different Scenarios
Source: PLoS One. 2008 Mar 12;3(3):e1790. doi: 10.1371/journal.pone.0001790 (PMC2258437; doi:10.1371/journal.pone.0001790)

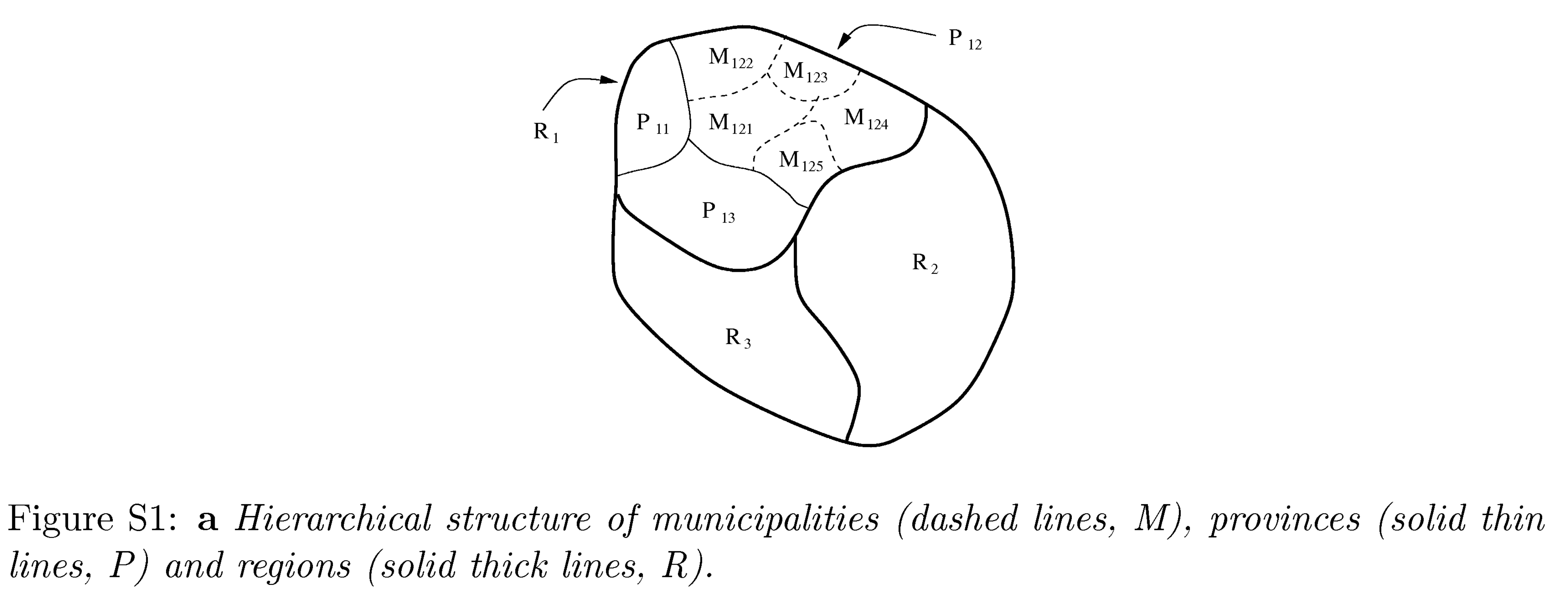

Supplement: Figure S1 — (0.30 MB TIF) [file pone.0001790.s007.tif]

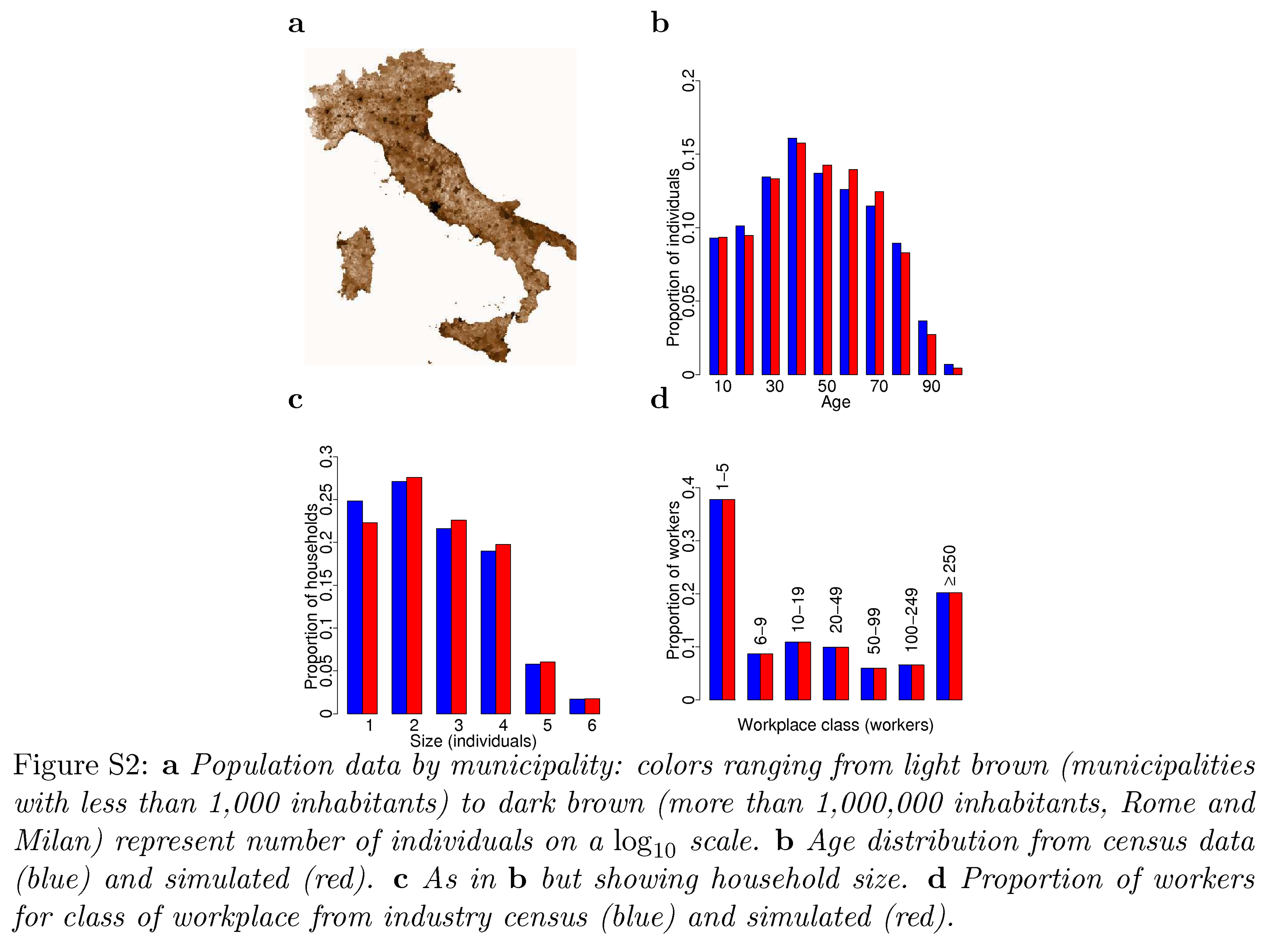

Supplement: Figure S2 — (1.56 MB TIF) [file pone.0001790.s008.tif]

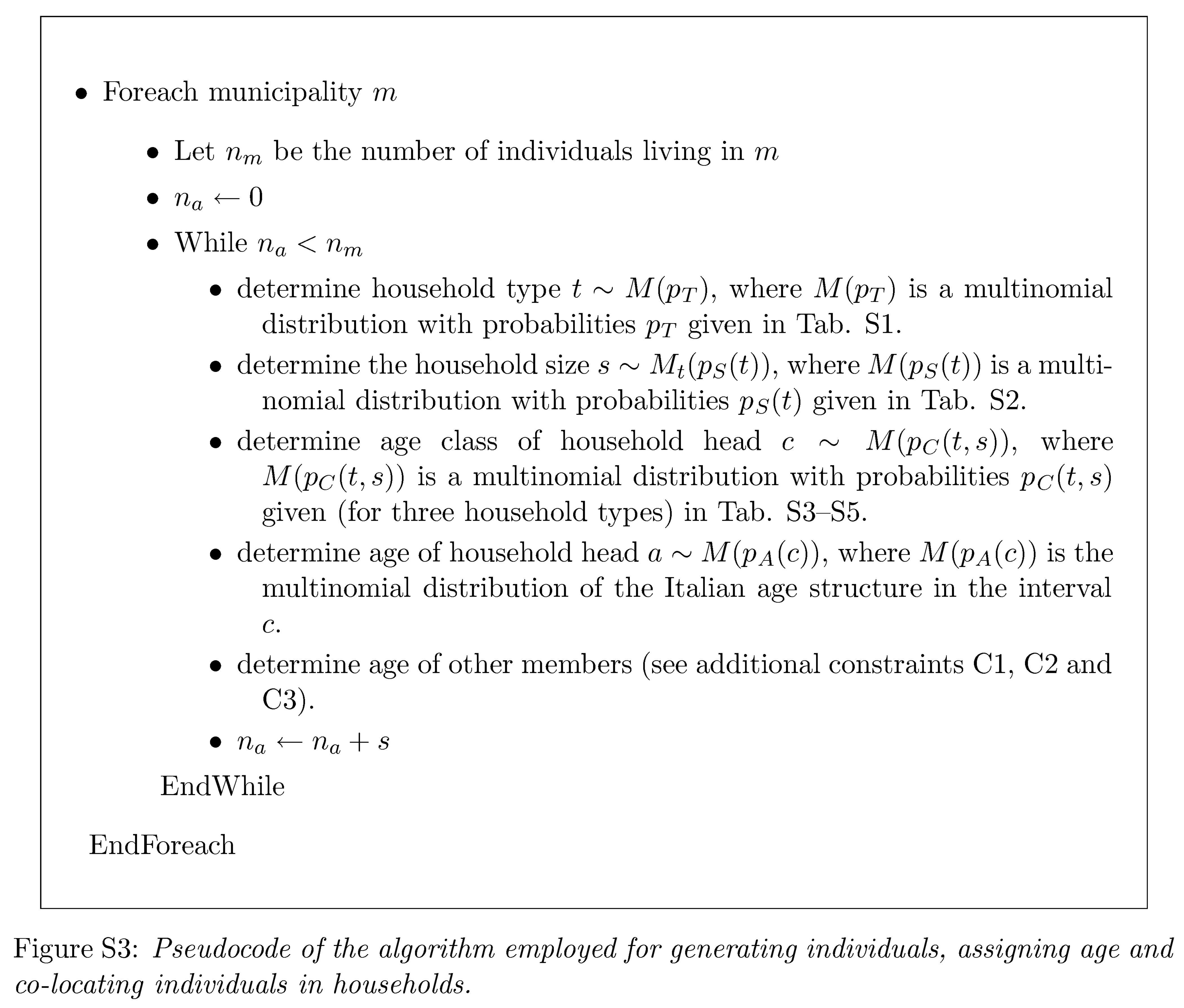

Supplement: Figure S3 — (1.05 MB TIF) [file pone.0001790.s009.tif]

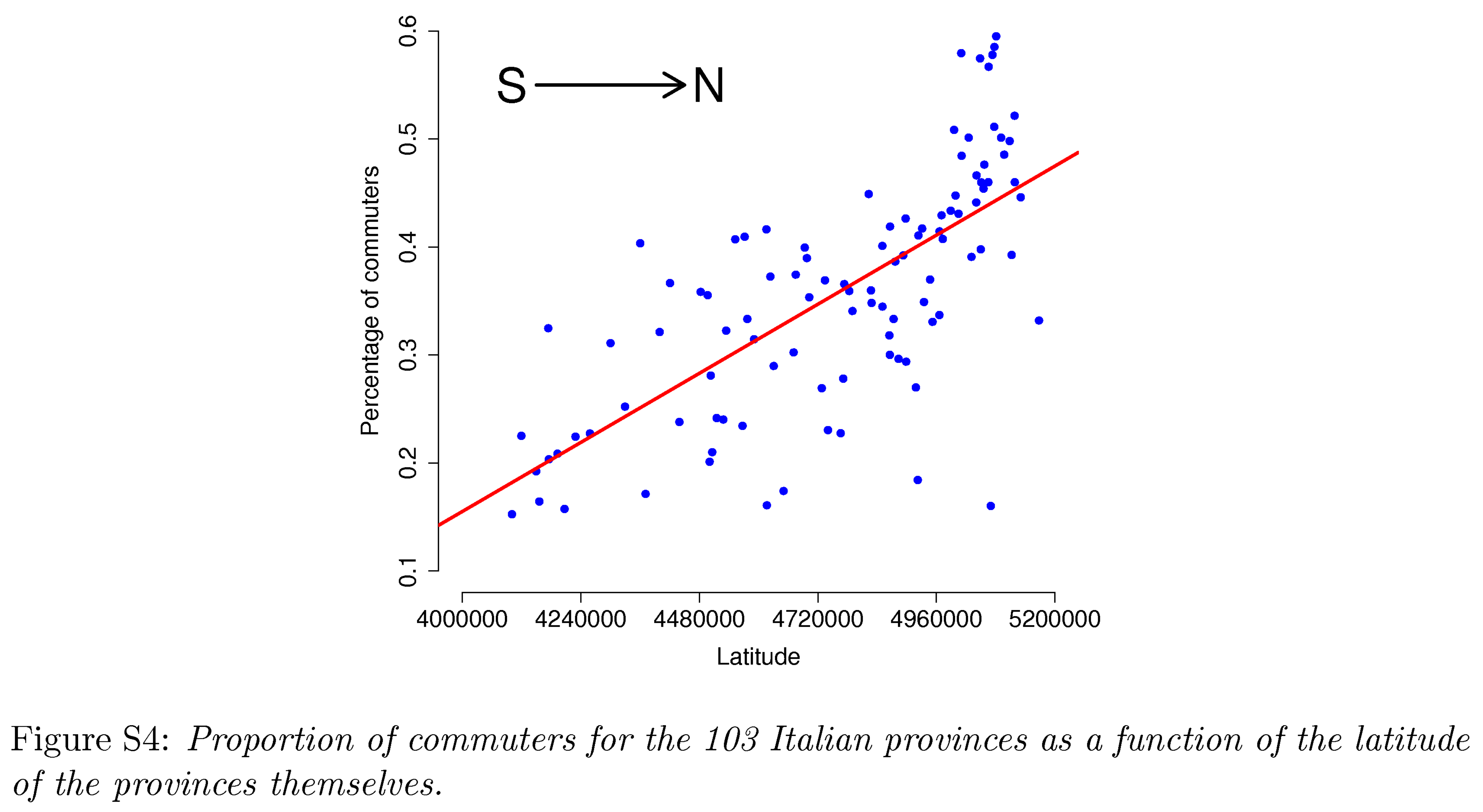

Supplement: Figure S4 — (0.38 MB TIF) [file pone.0001790.s010.tif]

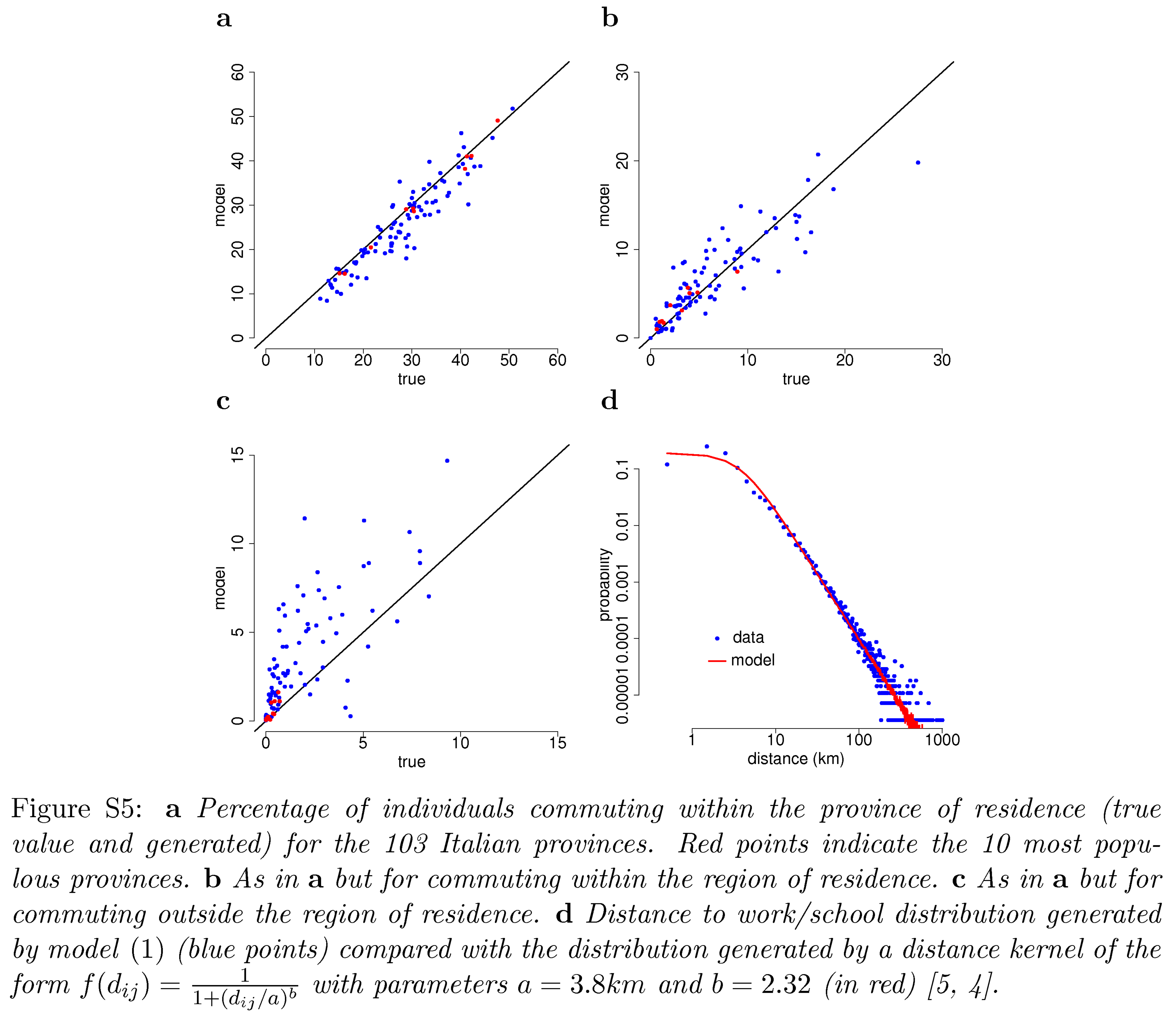

Supplement: Figure S5 — (0.98 MB TIF) [file pone.0001790.s011.tif]

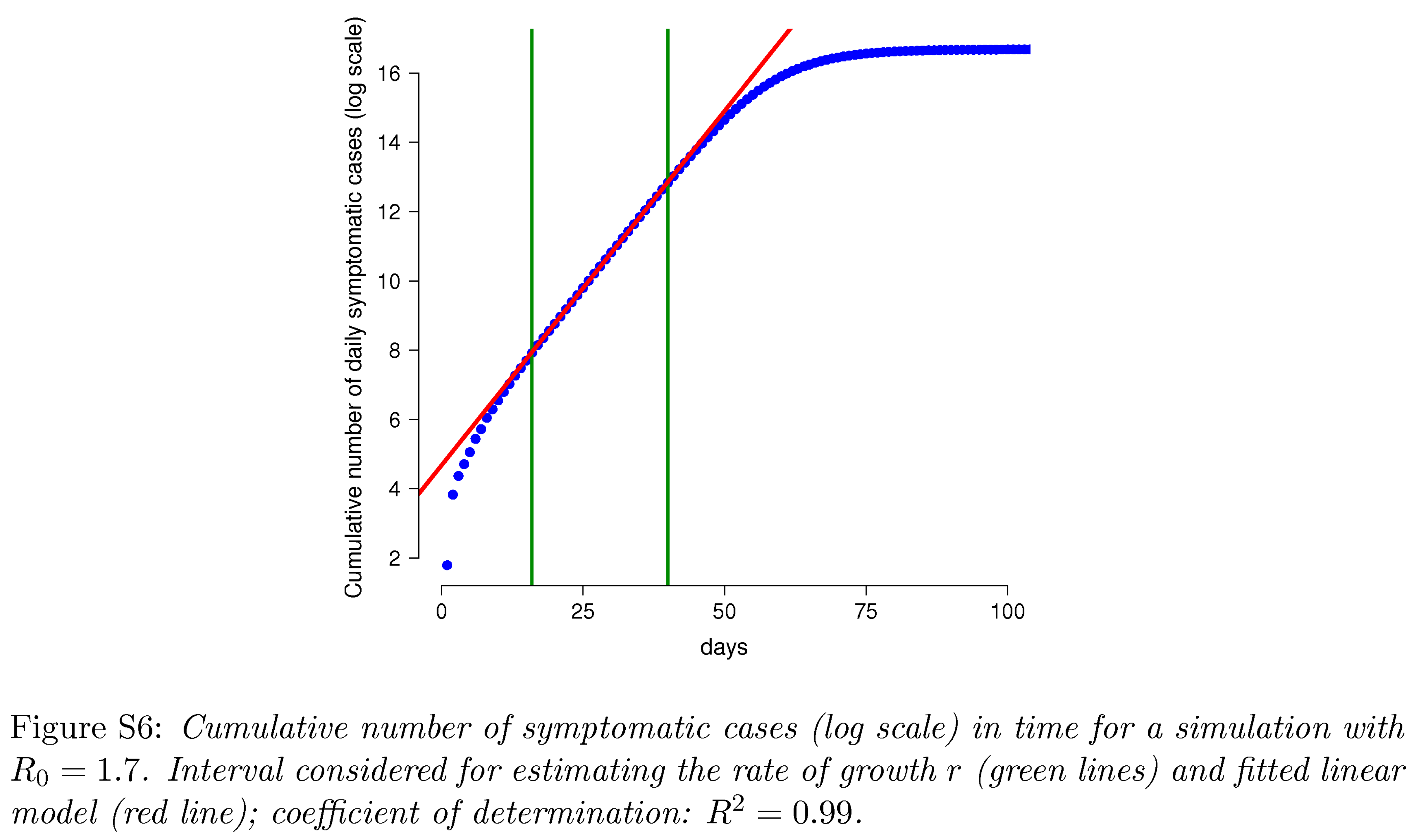

Supplement: Figure S6 — (0.51 MB TIF) [file pone.0001790.s012.tif]

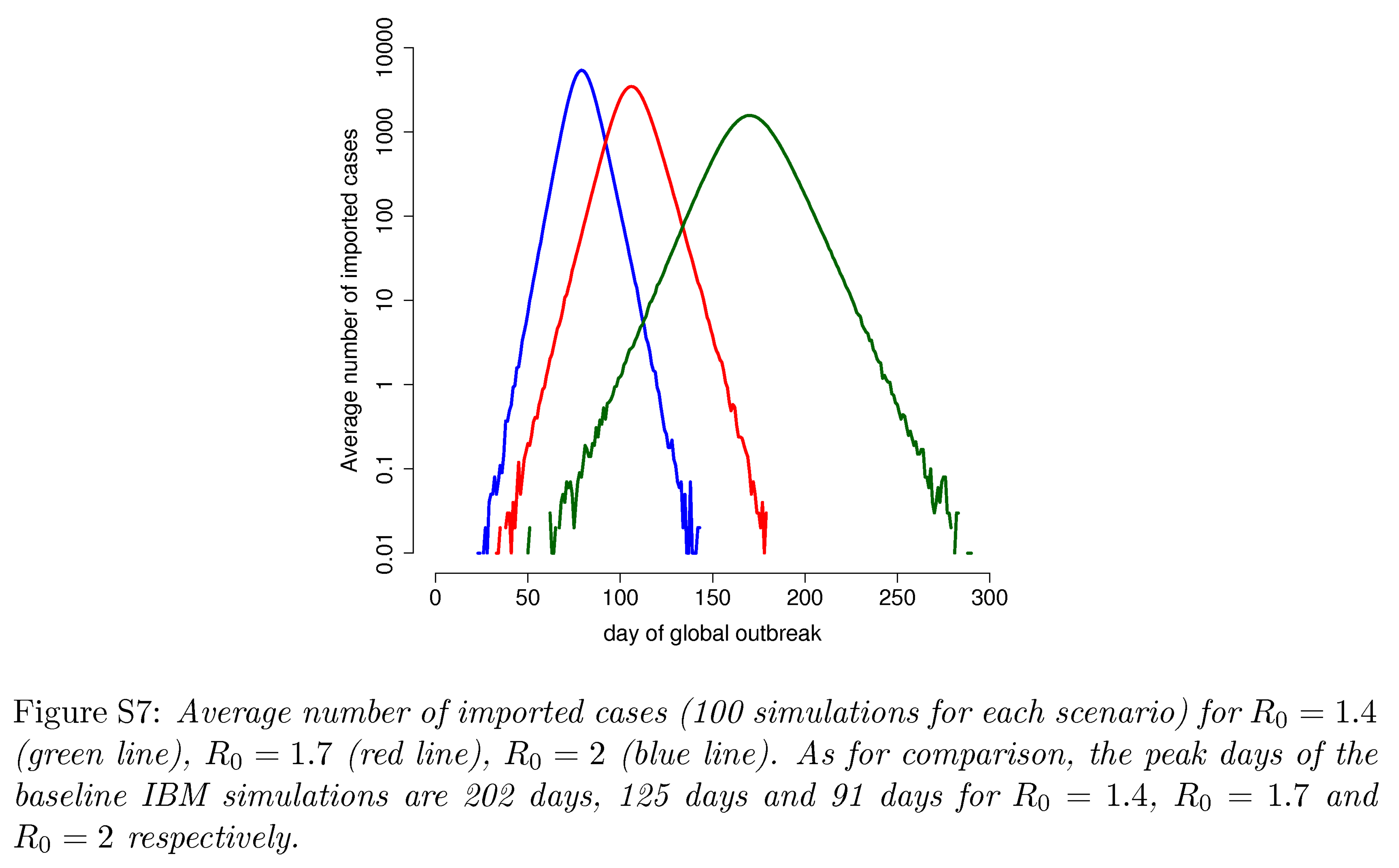

Supplement: Figure S7 — (0.62 MB TIF) [file pone.0001790.s013.tif]

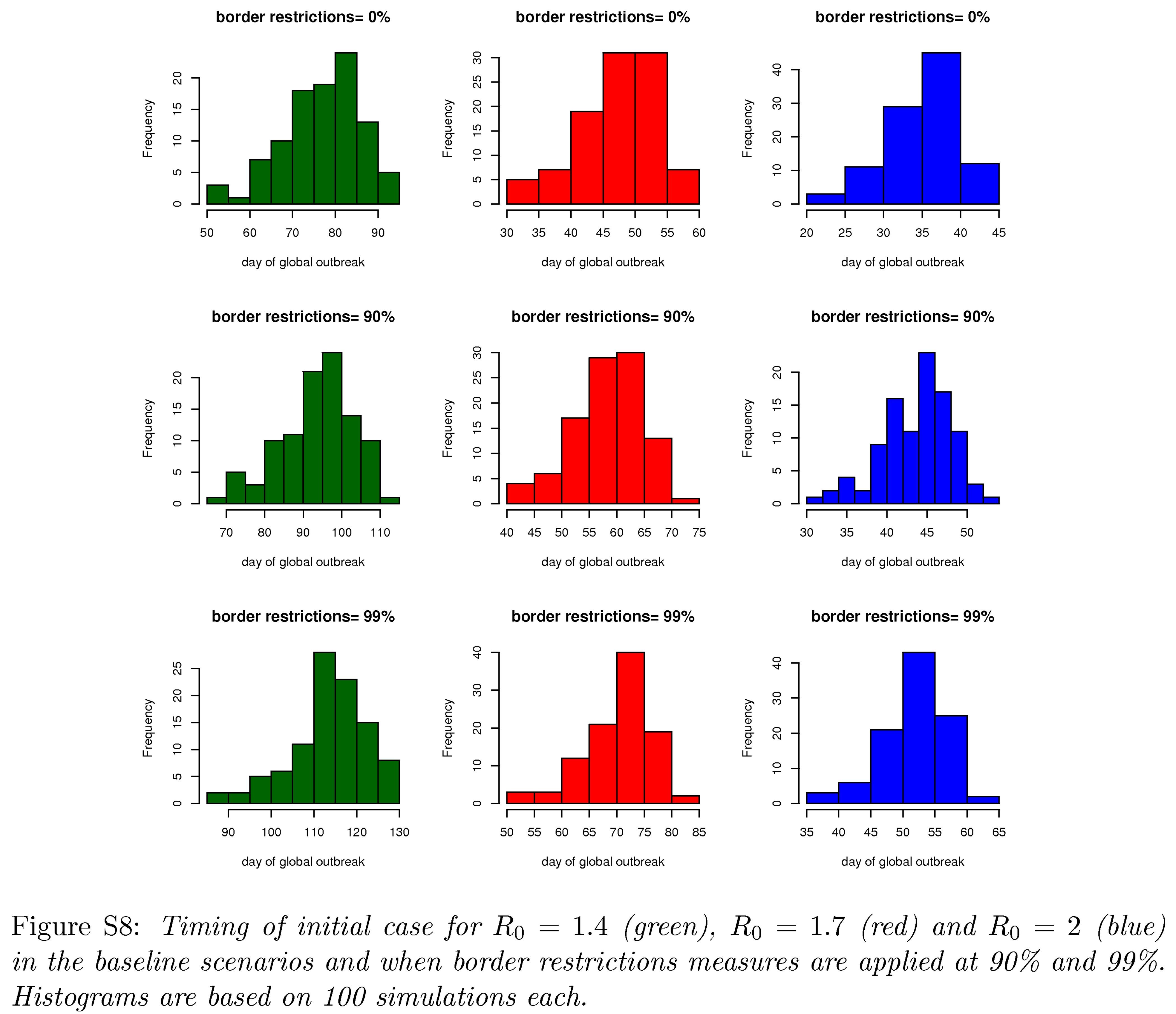

Supplement: Figure S8 — (0.93 MB TIF) [file pone.0001790.s014.tif]

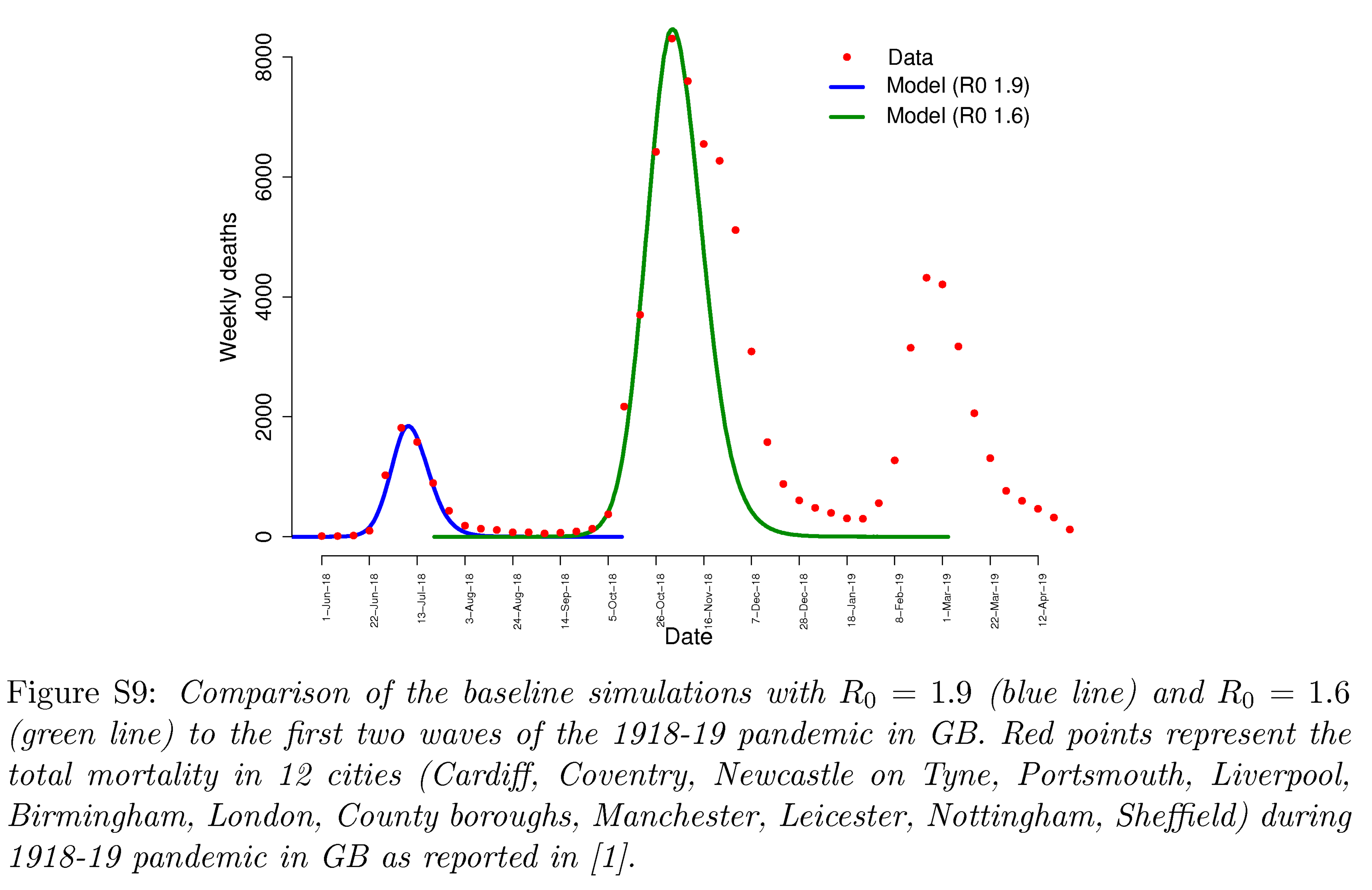

Supplement: Figure S9 — (0.72 MB TIF) [file pone.0001790.s015.tif]

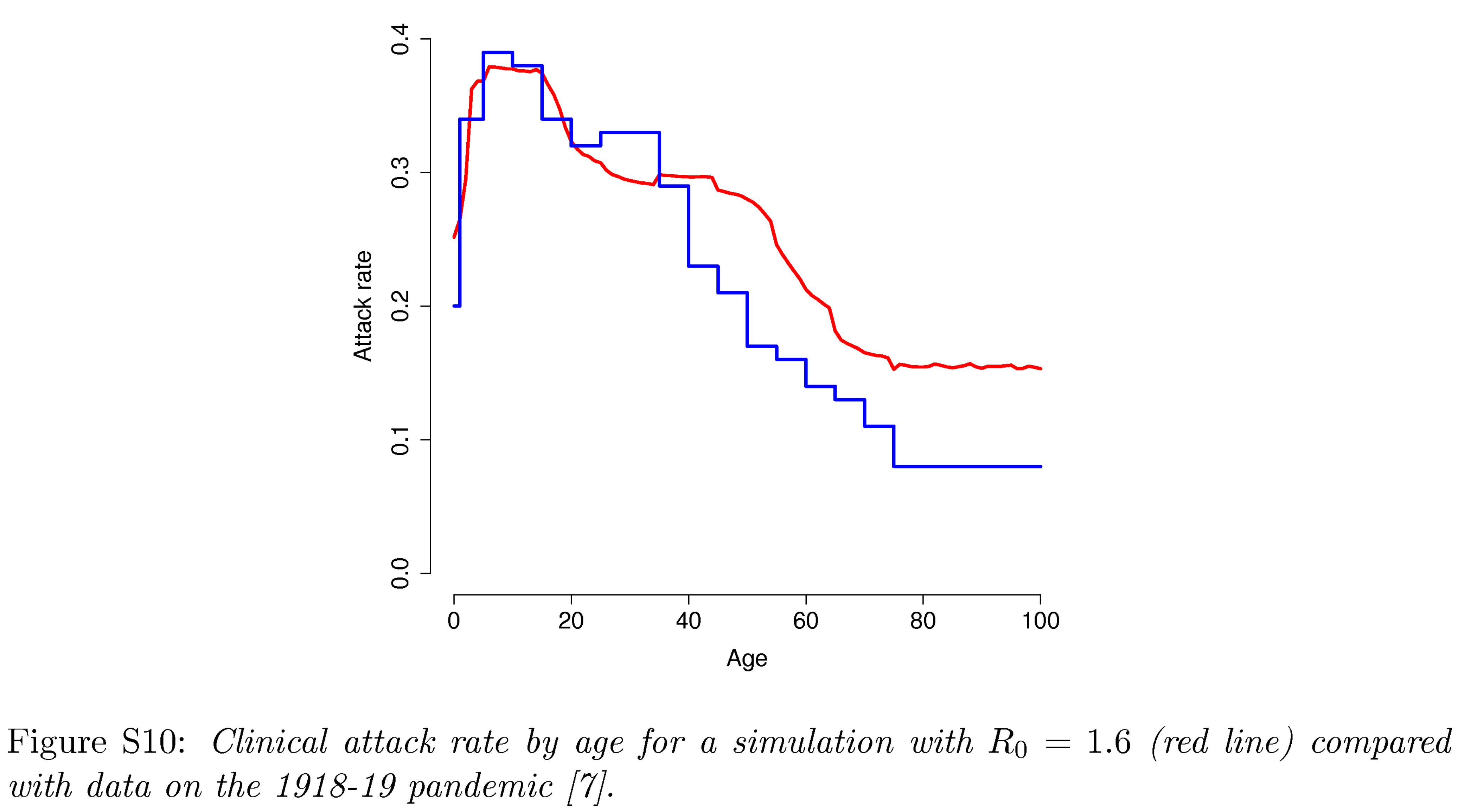

Supplement: Figure S10 — (0.30 MB TIF) [file pone.0001790.s016.tif]
